# Twelve-Month Results From the CISTO Study Comparing Radical Cystectomy Versus Bladder-Sparing Therapy for Recurrent High-Grade Non–Muscle-Invasive Bladder Cancer

**DOI:** 10.1200/JCO-25-01324

**Published:** 2025-12-15

**Authors:** John L. Gore, Erika M. Wolff, Michael G. Nash, Bryan A. Comstock, Scott M. Gilbert, Sam S. Chang, Stephanie Chisolm, Douglas B. MacLean, Jonathan L. Wright, Max R. Kates, Kamal S. Pohar, Thomas J. Guzzo, Trinity J. Bivalacqua, Kenneth G. Nepple, Jeffrey S. Montgomery, Kristen R. Scarpato, Solomon L. Woldu, Viraj A. Master, David Y.T. Chen, Matthew Mossanen, Siamak Daneshmand, Brock B. O'Neil, Mark D. Tyson, Mary E. Westerman, Ashish M. Kamat, Ahmed M. Mansour, Karim Chamie, Stephen B. Riggs, Janet B. Kukreja, Parth K. Modi, Tullika Garg, Charles C. Peyton, Jeffrey W. Nix, Rian Dickstein, Adam J. Gadzinski, Alex Sankin, Neal D. Shore, Brian R. Lane, Jeffrey C. Bassett, Sanjay Patel, David S. Morris, Liam C. Macleod, Eugene K. Lee, Chad R. Ritch, Kristin M. Follmer, Jenney R. Lee, Sung Min Kim, Larry G. Kessler, Angela B. Smith, on behalf of the CISTO Collaborative group, John L. Gore

**Affiliations:** ^1^ Department of Urology, University of Washington, Seattle, WA; ^2^ Department of Biostatistics, University of Washington, Seattle, WA; ^3^ Department of Genitourinary Oncology, H. Lee Moffit Cancer Center and Research Institute, Tampa, FL; ^4^ Department of Urology, Vanderbilt University Medical Center, Nashville, TN; ^5^ Bladder Cancer Advocacy Network, Bethesda, MD; ^6^ CISTO Advocate Advisory Board, Seattle, WA; ^7^ Department of Urology, The James Brady Urological Institute, Johns Hopkins University School of Medicine, Baltimore, MD; ^8^ Department of Urology, The Ohio State University, Columbus, OH; ^9^ Division of Urology, Department of Surgery, University of Pennsylvania Perelman School of Medicine, Philadelphia, PA; ^10^ Department of Urology, University of Iowa, Iowa City, IA; ^11^ Department of Urology, Michigan Medicine, Ann Arbor, MI; ^12^ Department of Urology, University of Texas Southwestern Medical Center, Dallas, TX; ^13^ Department of Urology and Winship Cancer Institute, Emory University, Atlanta, GA; ^14^ Department of Urology, Fox Chase Cancer Center, Philadelphia, PA; ^15^ Brigham and Women's Hospital, Boston, MA; ^16^ Department of Urology, Keck School of Medicine, University of Southern California, Los Angeles, CA; ^17^ Division of Urology, Huntsman Cancer Institute, University of Utah, Salt Lake City, UT; ^18^ Department of Urology, Mayo Clinic, Scottsdale, AZ; ^19^ Department of Urology, University of North Carolina at Chapel Hill, Chapel Hill, NC; ^20^ Department of Urology, University of Texas MD Anderson Cancer Center, Houston, TX; ^21^ Department of Urology, University of Texas Health, San Antonio, TX; ^22^ Department of Urology, David Geffen School of Medicine at UCLA, Los Angeles, CA; ^23^ Department of Urology, Levine Cancer Institute, Atrium Health, Charlotte, NC; ^24^ Department of Urology, University of Colorado, Denver, CO; ^25^ Section of Urology, Department of Surgery, University of Chicago, Chicago, IL; ^26^ Department of Urology, Geisinger Health System, Danville, PA; ^27^ Department of Urology, University of Alabama, Birmingham, AL; ^28^ Chesapeake Urology, Baltimore, MD; ^29^ Comprehensive Urology, Royal Oak, MI; ^30^ Department of Urology, Montefiore Medical Center and Albert Einstein School of Medicine, Bronx, NY; ^31^ Carolina Urologic Research Center, Myrtle Beach, SC; ^32^ Division of Urology, Corewell Health West, Grand Rapids, MI; ^33^ Department of Urologic Oncology, Hoag Memorial Presbyterian Hospital, Newport Beach, CA; ^34^ Department of Urology, University of Oklahoma Health Sciences Center, Oklahoma City, OK; ^35^ Urology Associates, PC, Nashville, TN; ^36^ Department of Urology and Heiman Cancer Center, Asante Rogue Regional Medical Center, Medford, OR; ^37^ Department of Urology, University of Kansas Medical Center, Kansas City, KS; ^38^ Desai Sethi Urology Institute, University of Miami Miller School of Medicine, Miami, FL; ^39^ Department of Health Systems and Population Health, School of Public Health, University of Washington, Seattle, WA

## Abstract

**PURPOSE:**

To compare patient-reported and clinical outcomes between radical cystectomy (RC) and bladder-sparing therapy (BST) in patients with recurrent high-grade non–muscle-invasive bladder cancer (NMIBC).

**PATIENTS AND METHODS:**

This pragmatic, prospective observational cohort study was designed with patients, who selected and prioritized outcomes. Eligible adults were candidates for both RC or BST, had previous induction Bacillus Calmette-Guérin (BCG), and received their last treatment within 12 months. The primary outcome was the EORTC-QLQ-C30 physical function scale at 12 months. Secondary outcomes included other EORTC-QLQ-C30 scales, depression, anxiety, bladder cancer–specific quality of life (QOL), financial burden, and cancer-specific outcomes. Targeted maximum likelihood estimation (TMLE) was used to calculate average treatment effect (ATE) estimates between arms. Inverse probability weighted risk ratios (wRR) were calculated using quasi-Poisson regression.

**RESULTS:**

Of 570 participants (mean age 71.4 years; 21% female), 371 selected BST and 199 selected RC. Physical function was significantly worse in the RC arm at 3 months; by 9 months, there was no difference between arms, and at 12 months, physical function did not differ (ATE, 0.9; 95% CI, –0.6 to 2.4; *P* = .22). RC was associated with better emotional function, generic health-related QOL, and financial burden, and lower depression and anxiety, while BST was associated with better bowel and sexual health. Cancer-specific survival was 99% for BST versus 96% for RC (wRR, 0.99; 95% CI, 0.97 to 1.01). RC was associated with a higher risk of adverse events and serious adverse events, including a 90-day mortality rate of 2.5%.

**CONCLUSION:**

Most patient-prioritized outcomes were similar or better among participants who chose RC compared with BST. These findings support the continued role of RC in managing recurrent high-grade NMIBC.

## INTRODUCTION

Bladder cancer is the fifth most common cancer in the United States.^1^ Most patients (74%) are diagnosed with non–muscle-invasive bladder cancer (NMIBC),^2^ with the oldest median age at diagnosis, intensive surveillance requirements,^3,4^ high recurrence and progression rates, and one of the highest lifetime treatment costs of all cancers.^3,5-7^

**CONTEXT**

**Key Objective**
How do patient-reported and clinical outcomes compare between bladder-sparing therapy (BST) and radical cystectomy (RC) for recurrent high-grade non–muscle-invasive bladder cancer (NMIBC)?
**Knowledge Generated**
In this prospective observational study of 570 patients, there was no significant difference in 12-month physical function between treatments, and cancer-specific survival was similar. RC was associated with better emotional well-being and lower financial burden, while BST was associated with better bowel and sexual function as well as a lower risk of adverse events and serious adverse events.
**Relevance *(A. Necchi)***
The CISTO study adds important information to the existing literature, representing a call for additional studies focused on NMIBC. Based on these findings, the role of radical cystectomy and bladder-saving strategies and their impact on quality-of-life outcomes need further discussion in patient-centric strategies.*
*Relevance section written by *JCO* Associate Editor Andrea Necchi, MD.

High-grade NMIBC is initially treated with endoscopic resection and intravesical therapy, most commonly Bacillus Calmette-Guérin (BCG),^2,3,8^ but recurs in 24%-61% of patients within 12 months.^9^ For recurrent high-grade NMIBC, guidelines recommend either bladder-sparing therapy (BST) with additional intravesical therapy or intravenous immunotherapy or bladder removal with radical cystectomy (RC).^7,10^ Decision making involves weighing the risk of progression of bladder cancer and loss of a window of potential cure versus the risk of morbidity and impact on daily life with bladder removal. Yet contemporary evidence provides limited support for the patient-centered outcomes underlying this decision.^11^ No previous studies have compared RC with BST using both clinical and patient-reported outcomes. Thus, patients and clinicians navigate substantial uncertainty in managing recurrent high-grade NMIBC.

To address this evidence gap, we partnered with patients to design and conduct a comparative effectiveness study that assessed outcomes selected and prioritized by patients using a prospective observational design, chosen because of low patient willingness (11%) to consent to random assignment.^12-14^ The Comparison of Intravesical Therapy and Surgery as Treatment Options (CISTO) study (ClinicalTrials.gov identifier: NCT03933826) is a pragmatic cohort study of patients with recurrent high-grade NMIBC eligible for both RC and BST.^12,14^ We report the primary outcome of 12-month physical functioning and select secondary outcomes. The primary study hypothesis, given the morbidity of RC, was that patients undergoing RC would have worse physical functioning at 12 months than those receiving BST.

## METHODS

Consecutive patients were approached at 36 academic and community urology practices across the United States. Vanderbilt University's Institutional Review Board (IRB) was the single IRB for most sites; select sites used their institutional IRB. All participants provided informed consent. Participants were enrolled after completing a baseline survey and selecting their treatment. The study was funded by the Patient-Centered Outcomes Research Institute (PCORI, PCS-2017C3-9380); the funders had no role in the design of the trial; in the collection, analysis, or interpretation of the data; or in the reporting of the results.

### Study Population

Adults with recurrent high-grade NMIBC (tumor stage Tis, Ta, or T1) who were candidates for both RC or BST, had at least attempted previous induction intravesical BCG, and whose last NMIBC treatment occurred within the past 12 months were eligible (Data Supplement, Tables S1 and S2). Exclusions included being medically ineligible for either treatment arm per the treating physician, previous MIBC or upper urinary tract urothelial cancer, or planned participation in a phase I or phase II interventional NMIBC trial.

### Study Outcomes

The primary outcome was 12-month patient-reported physical function, measured with the European Organisation for Research and Treatment of Cancer Core Quality of Life (EORTC QLQ-C30) physical functioning scale.^15^ This was selected as the primary outcome on the basis of patient and caregiver input.^12,14^ Physical function was compared between treatment arms in the prespecified subgroups of males versus females, age ≥75 years versus <75 years, partnered versus unpartnered, and concomitant carcinoma in situ (CIS) versus no CIS. Concomitant CIS was selected, given lower response rates to BST and contemporary clinical trials that separately analyze the outcomes of this group.^9,16-18^ All patient-reported outcomes were assessed from the time of enrollment.

Secondary EORTC QLQ-C30 scales included global health status, role, emotional, cognitive, and social functioning, and the symptoms financial difficulties, fatigue, nausea, pain, dyspnea, insomnia, appetite loss, constipation, and diarrhea.^15^ Scores range from 0 to 100, with higher function scores indicating better health and higher symptom scores indicating more symptoms.

Depression and anxiety were measured with Patient-Reported Outcome Measurement Information System (PROMIS-29) depression and anxiety domains. Scores normalize to a mean of 50 and standard deviation (SD) of 10; higher scores indicate greater symptoms.^19^ Generic health-related QOL was assessed with the EuroQoL EQ-5D-5L (EQ-5D); scores range from 0 to 1 and higher scores indicate better health.^20^ Financial toxicity was measured with the Comprehensive Score for Financial Toxicity (COST),^21^ which includes questions about concern with covering the costs of treatment, out-of-pocket expenses, ability to work, and impact and worry about finances. COST scores range from 0 to 44, with lower scores indicating greater financial distress. Bladder cancer–specific QOL was assessed with the Bladder Cancer Index (BCI), which can be used in both RC and BST populations, with function and bother urinary, bowel, and sexual health subdomains.^22^ The questions are framed to allow patients with their native bladder, with a neobladder, and with an ileal conduit urinary diversion to answer the same urinary function and bother questions; scores range from 0 to 100, with higher scores indicating better quality of life (QOL). EORTC QLQ-C30, BCI, and PROMIS measures were also collected 3, 6, and 9 months after enrollment.

Secondary clinical outcomes included 12-month recurrence-free, progression-free, metastasis-free, cancer-specific, and overall survival (OS) assessed via electronic health record (EHR) abstraction (Data Supplement, Table S3). All clinical outcomes were assessed within 365 days from the diagnostic transurethral resection of bladder tumor establishing recurrent high-grade NMIBC. For recurrence, progression, and metastasis events, we considered participants to be event-free for 90 days after a surveillance procedure with negative findings as another procedure would not be indicated during this period. Vital status was determined on the basis of date of last survey completion or last clinical encounter, with obituary searches for missing participants.

Adverse events (AEs) and serious adverse events (SAEs) were categorized by grade and the National Cancer Institute's Common Terminology Criteria for Adverse Events (CTCAE) version 5.0.^23^ SAEs were defined as death, a life-threatening event, inpatient hospitalization (other than for RC in the RC arm), and prolonged hospitalization after RC (≥14 days on the basis of previously published thresholds).^24,25^ We defined cancer-related SAEs by attribution to bladder cancer or its treatment.

### Statistical Analysis

All analyses were performed with R version 4.3.2 (R Foundation for Statistical Computing).^26^ A sample size of 572 patients was selected to provide >80% power to detect small differences between treatments (5.5 points in EORTC-QLQ-C30 physical function) or moderate clinically important differences within subgroups as small as 30% of the cohort (9.9 points in EORTC-QLQ-C30 physical function) in either direction.^14^

Patient data were analyzed with an intention-to-treat (ITT) framework for the primary analysis, where treatment arm was decided upon at enrollment regardless of whether that treatment was initiated. Targeted maximum likelihood estimation (TMLE) was used as the primary analytic approach to mitigate treatment selection bias and confounding (tmle R package).^27-29^ TMLE is a modern analytic method for causal effect estimation using observational data that combines machine learning with traditional regression modeling to produce estimates that are both robust and efficient. TMLE is doubly robust, meaning that it can provide valid estimates even if either the treatment model or the outcome model is mis-specified because of an omitted confounding variable. Unlike propensity score–based approaches that focus on creating carefully matched treatment and control groups, TMLE allows for the inclusion of all patient participants and their reported outcomes without the need to discard unmatched observations.

We first created covariate-adjusted linear mixed-effects regression models to generate an initial estimate of the treatment effect of RC by comparing predicted outcomes for each patient under the assumption that they had been treated with RC and as if they had been treated with BST (see eTable 4 for specific variables included). We next estimated the probability of treatment with RC using super learning, an ensemble machine learning method that optimally combines multiple predictive algorithms, with the following base learners (SuperLearner R package): logistic regression with and without stepwise selection of covariates, generalized additive models, Bayesian generalized linear models, elastic net regression, random forests, gradient descent boosting, and discrete Bayesian additive regression trees.

We used these probabilities to update the initial estimate of each patient's pair of potential outcomes in a step known as targeting, which improves bias reduction by aligning the estimate with the observed treatment assignment. The final TMLE estimate, the average treatment effect (ATE), is interpreted as the causal mean difference in outcomes if all patients had been treated with RC versus having been treated with BST.^30^ Sensitivity analyses used generalized estimating equations (GEE) with our full sample and TMLE with as-treated participants, defined as having received treatment within 6 months of enrollment.

For secondary clinical outcomes, we generated inverse probability-weighed risk ratios (wRR) with quasi-Poisson regression using the “svyglm” function from the survey package in R, including all participants with at least 1 day of exposure and incorporating the log of exposure time as an offset.^31-33^ We also created Kaplan-Meier curves stratified by treatment arm. Two-sided 95% CIs were used for inference without adjustment for multiple survival end points. Events of unknown attribution were conservatively classified as cancer-related as a primary approach and excluded in sensitivity analyses.

We performed tenfold multiple imputation (MICE R package) for all missing outcomes and covariates for analyses of all patient-reported outcomes (Data Supplement, Tables S5 and S6).^34^ Fully observed baseline variables were set to predictor-only and were not imputed; baseline characteristics were reported from observed data. We prespecified a two-sided alpha of .05 for statistical significance of the primary outcome. For secondary outcomes, we present unadjusted 95% CI.

### Ethics Approval and Consent to Participate

The CISTO study protocol was approved by the Vanderbilt University Medical Center's IRB on April 17, 2019 (VUMC IRB #190791), and protocol version seven was approved on May 22, 2023. All participants provided written informed consent.

## RESULTS

### Participant Characteristics

**FIG 1.**
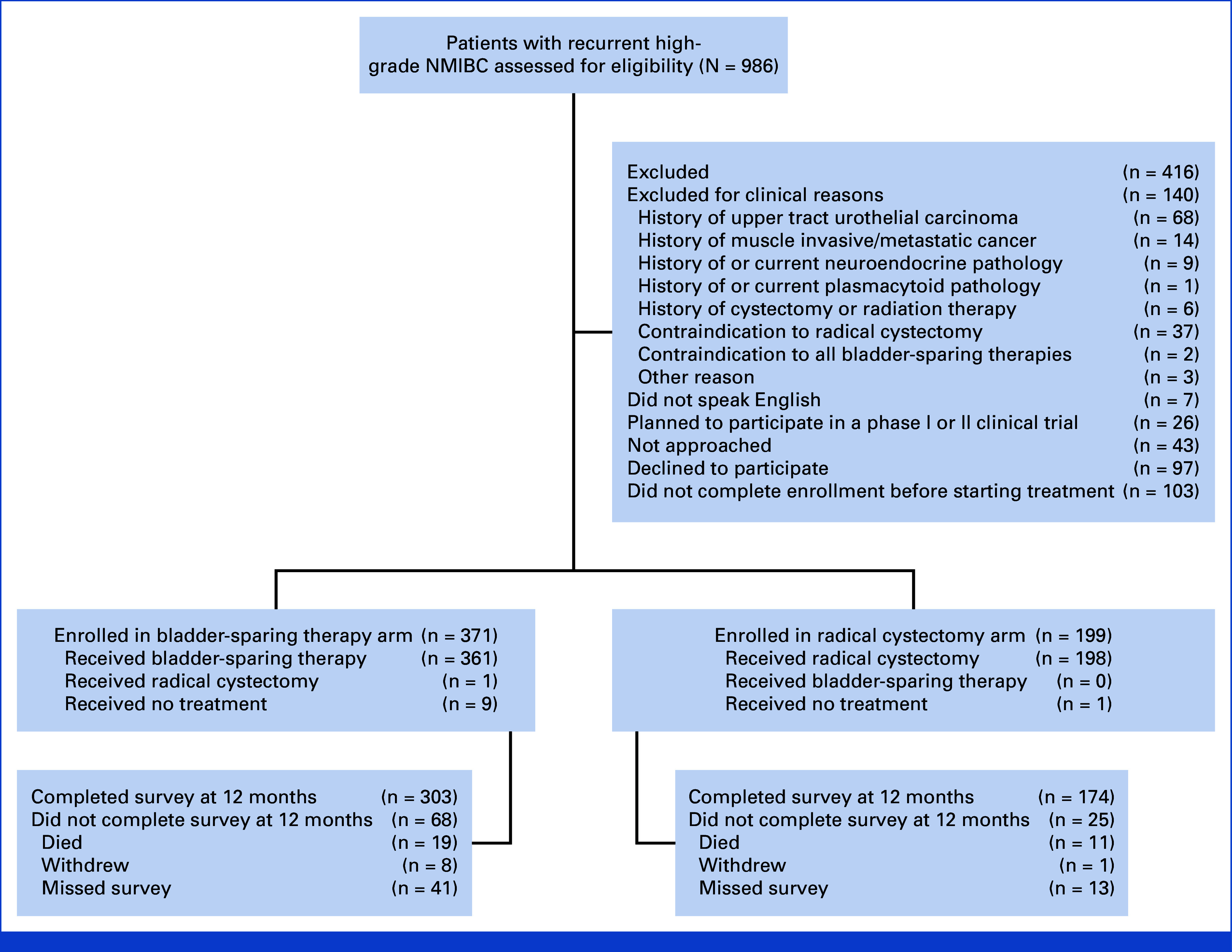
Screening, enrollment, treatment selection, and follow-up for the CISTO study. CISTO, Comparison of Intravesical Therapy and Surgery as Treatment Options; NMIBC, non–muscle-invasive bladder cancer.

Between July 2019 and December 2023, 570 patients were enrolled; 371 selected BST and 199 selected RC (Fig 1). Participants had mean age 71.4 years (SD, 8.7), 21% were female, 92% were White, 5% were Black, and 3% were Hispanic (Data Supplement, Table S7). Among participants who had not withdrawn or passed away within the survey window, 90% completed the 12-month survey.

Patient characteristics were similar between study arms with respect to sex, race and/or ethnicity, education, employment status, number of household members, household income, health insurance, smoking history, comorbidity burden, general QOL, and years since initial NMIBC diagnosis (Table 1, Data Supplement, Table S8). Participants in the RC arm were younger, less likely to be partnered or reside in urban areas, and more likely to have someone who helps with medical needs. Participants in the RC arm had worse urinary and bowel health at enrollment and reported greater anxiety. The presence of concomitant CIS, stage classification T1 NMIBC, secondary nonurothelial histology, and recent treatment with a BST that was not BCG was more common in the RC arm.

**TABLE 1.** Characteristics of Participants in the Comparison of Intravesical Therapy and Surgery as Treatment Options Study

Characteristic	BST	RC	Missing No.	*P*
Patient characteristics, No.	371	199		
Age, years, mean (SD)	72.4 (8.3)	69.6 (9.1)	0	<.001
≥75 years, No. (%)	150 (40)	62 (31)	0	.03
Female gender, No. (%)	78 (21)	39 (20)	0	.74
Non-Hispanic White race/ethnicity, No. (%)	332 (89)	182 (91)	0	.56
Has a spouse or partner, No. (%)	288 (78)	170 (86)	5	.03
Employed, No. (%)	100 (27)	52 (26)	0	.92
Retired, No. (%)	238 (64)	128 (64)	0	1.00
Private health insurance, No. (%)	119 (32)	76 (38)	2	.14
Medicare, No. (%)	267 (72)	136 (69)	2	.38
Urban dweller, No. (%)^a^	323 (87)	156 (78)	0	.008
National Area Deprivation Index,^b^ median (IQR)	37.0 (21.0-61.0)	38.0 (19.5-61.5)	0	.95
Modified Elixhauser comorbidity index, mean (SD)	1.4 (5.0)	1.1 (4.5)	1	.50
Ever smoker, No. (%)	247 (67)	126 (63)	0	.46
Current smoker, No. (%)	28 (7.5)	10 (5.0)	0	.29
Tumor characteristics, No. (%)				
Carcinoma in situ	165 (45)	108 (55)	4	.02
Clinical T stage T1	110 (30)	113 (57)	0	<.001
Secondary variant histology	8 (2.2)	13 (6.6)	3	.01
Pathologic T stage at RC^c^				
T0	NA	31 (16)	NA	NA
Ta	NA	24 (12)	NA	NA
Tis	NA	62 (31)	NA	NA
T1	NA	37 (19)	NA	NA
T2	NA	24 (12)	NA	NA
T3	NA	18 (9.0)	NA	NA
T4a	NA	3 (1.5)	NA	NA
Patient-reported outcomes at enrollment, mean (SD)				
EORTC physical functioning^d^	88.6 (16.9)	89.8 (14.5)	0	.37
PROMIS depression^e^	48.1 (8.2)	49.0 (8.0)	5	.21
PROMIS anxiety^e^	51.7 (9.3)	54.0 (8.8)	5	.005
EQ-5D^d^	0.84 (0.13)	0.84 (0.12)	33	.61
BCI urinary summary^d^	84.9 (15.5)	77.7 (19.7)	5	<.001
BCI bowel summary^d^	80.9 (9.2)	78.7 (9.4)	6	.008
BCI sexual summary^d^	49.3 (25.5)	48.2 (26.6)	70	.66

Abbreviations: ADI, area deprivation index; BCI, Bladder Cancer Index; BST, bladder-sparing therapy; EORTC, European Organisation for Research and Treatment of Cancer Core QLQ-C30; EQ-5D, EuroQoL EQ-5D-5L; NA, not applicable; PROMIS, Patient-Reported Outcome Measurement Information System; QOL, quality of life; RC, radical cystectomy; RUCA, Rural-Urban Commuting Area; SD, standard deviation.

^a^ Based on ZIP code and RUCA codes.^35^

^b^ Based on ZIP code and the 2022 ADI v4.0.^36^

^c^ Stage from the RC specimen.

^d^ Higher score means better functioning/health/QOL.

^e^ Higher score means worse functioning/health/QOL.

### Treatment Characteristics

Among participants who selected BST, 109 (29%) received additional intravesical BCG, 200 (54%) received combination intravesical gemcitabine and docetaxel, 27 (7.3%) received intravenous pembrolizumab, 18 (5%) received other therapies, and 17 (5%) received no additional treatment within 1 year of enrollment (Data Supplement, Table S9). Among participants who selected RC, mean time to surgery was 2.8 months (SD, 1.6 months), 99 (51%) underwent robotic surgery, and 158 (79%) underwent an ileal conduit urinary diversion (Data Supplement, Table S9).

### Study Outcomes

**FIG 2.**
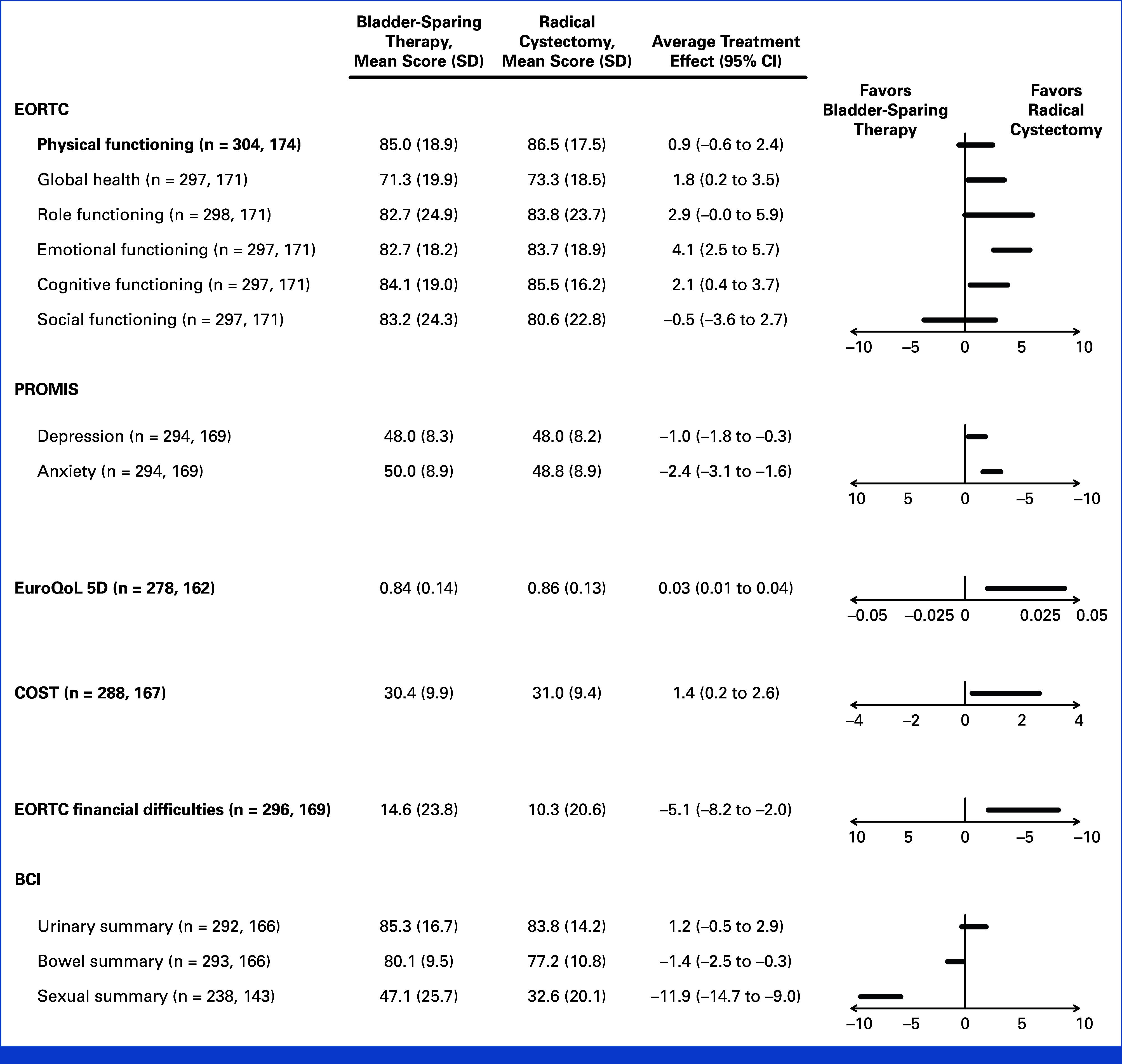
Standardized ATEs comparing BST and RC for recurrent high-grade non–muscle-invasive bladder cancer. Unadjusted domain and scale mean scores and standard deviations at 12 months after enrollment are shown for patients in the BST and RC arms who completed each measure. Adjusted ATEs of RC versus BST for each outcome are shown, with missing data imputed as described in the methods. ATE, average treatment effect; BCI, Bladder Cancer Index; BST, bladder-sparing therapy; COST, Comprehensive Score for Financial Toxicity; EORTC, European Organisation for Research and Treatment of Cancer; PROMIS, Patient-Reported Outcome Measurement Information System; RC, radical cystectomy; SD, standard deviation.

At 12 months, the primary outcome of EORTC QLQ-C30 physical function did not differ between the BST arm and the RC arm (mean 85.4 [SD, 17.7] *v* 86.2 [17.6], respectively; ATE, 0.9 [95% CI, –0.6 to 2.4]; *P* = .22; Fig 2, Data Supplement, Table S10). Sensitivity analyses revealed similar results (as-treated ATE, 1.1 [95% CI, –0.5 to 2.7]; GEE ATE, 1.0 [95% CI, –1.8 to 3.8]). In prespecified subgroups, RC was associated with slight but statistically significantly greater physical function compared with BST at 12 months among unpartnered patients (ATE, 3.3 [95% CI, 0.1 to 6.4]) and those with concomitant CIS (ATE, 2.7 [95% CI, 0.8 to 4.7], Data Supplement, Fig S1).

Emotional function, cognitive function, global health, anxiety, and depression were significantly better in the RC arm 12 months after enrollment (Fig 2). There were no significant differences between treatment arms in role function and social function. Participants in the BST arm reported significantly higher financial toxicity and financial difficulties 12 months after enrollment than participants in the RC arm. There were no significant differences in urinary summary score or urinary bother score by treatment arm, but urinary function score was significantly better in the RC arm at 12 months (Fig 2, Data Supplement, Fig S1). Participants in the RC arm had significantly worse 12-month bowel and sexual summary scores than those in the BST arm. Sensitivity analyses using GEE yielded similar results (Data Supplement, Table S11). Item missingness, highest for sexual function outcomes, is provided in the Data Supplement (Table S5).

**FIG 3.**
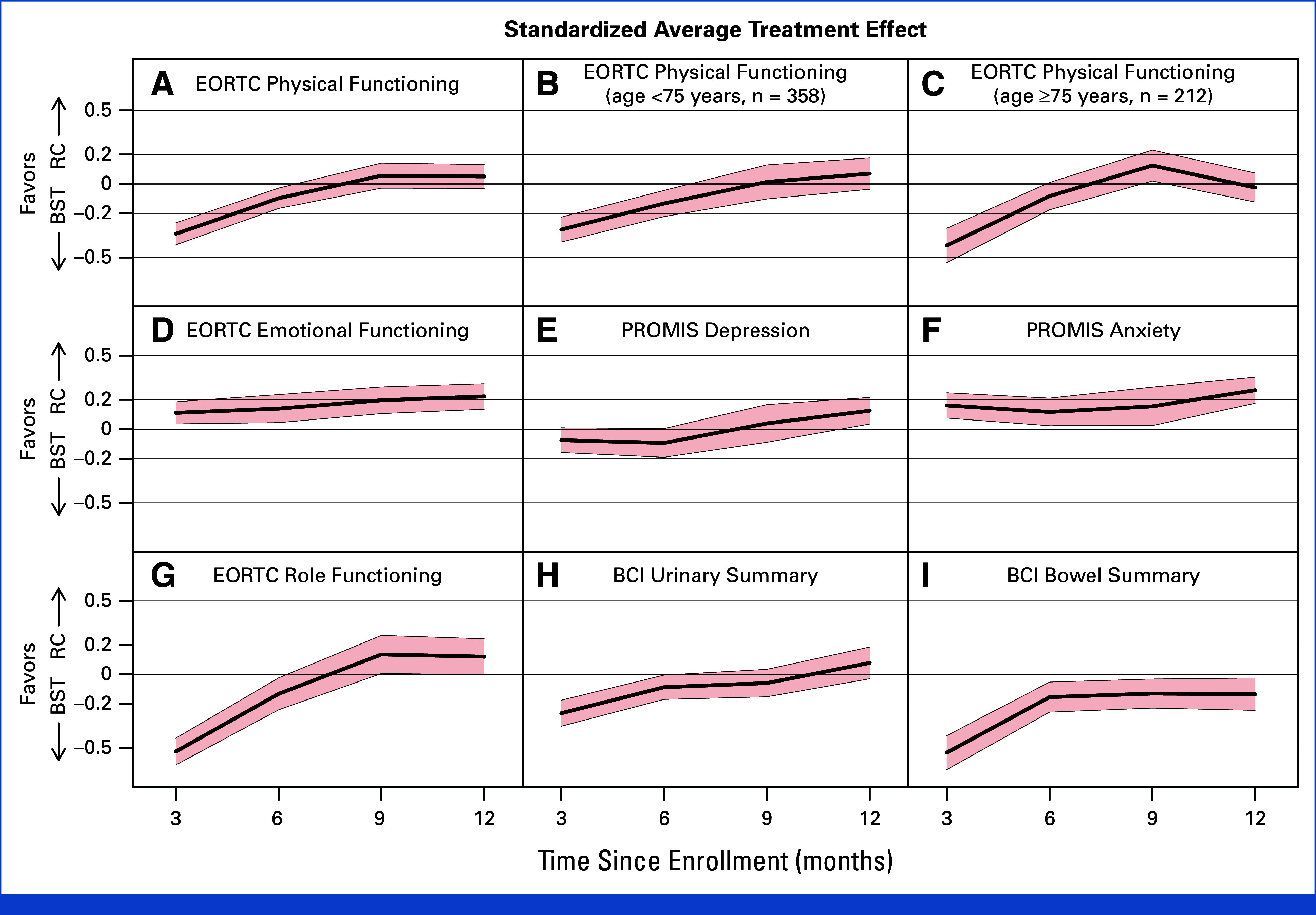
Standardized average treatment effect (95% CI) of longitudinal outcomes comparing BST and RC for recurrent high-grade non–muscle-invasive bladder cancer: EORTC QLQ-C30 physical function scale in (A) all participants and in (B) those younger than 75 years and (C) those age 75 years and older, (D) EORTC QLQ-C30 Emotional Function scale, PROMIS (E) Depression and (F) Anxiety scales, (G) EORTC QLQ-C30 role function scale, and BCI (H) Urinary and (I) Bowel summary scores in the 12 months after enrollment. BCI, Bladder Cancer Index; BST, bladder-sparing therapy; EORTC QLQ-C30, European Organisation for Research and Treatment of Cancer Core Quality of Life; PROMIS, Patient-Reported Outcome Measurement Information System; RC, radical cystectomy.

Adjusted longitudinal treatment effects for EORTC QLQ-C30, PROMIS, and BCI domains are displayed in Figure 3. EORTC QLQ-C30 physical and role function were significantly worse in the RC arm at 3 months compared with the BST arm, but no significant treatment effects were observed by 9 months, including for physical function among participants age 75 years and older. Emotional function and anxiety were better in the RC arm at all follow-up time points. Urinary and bowel health were significantly worse in the RC arm at 3 months. By 12 months, there were no significant differences between the two treatments with respect to urinary health, but bowel health remained worse in the RC arm.

### Clinical Outcomes

**FIG 4.**
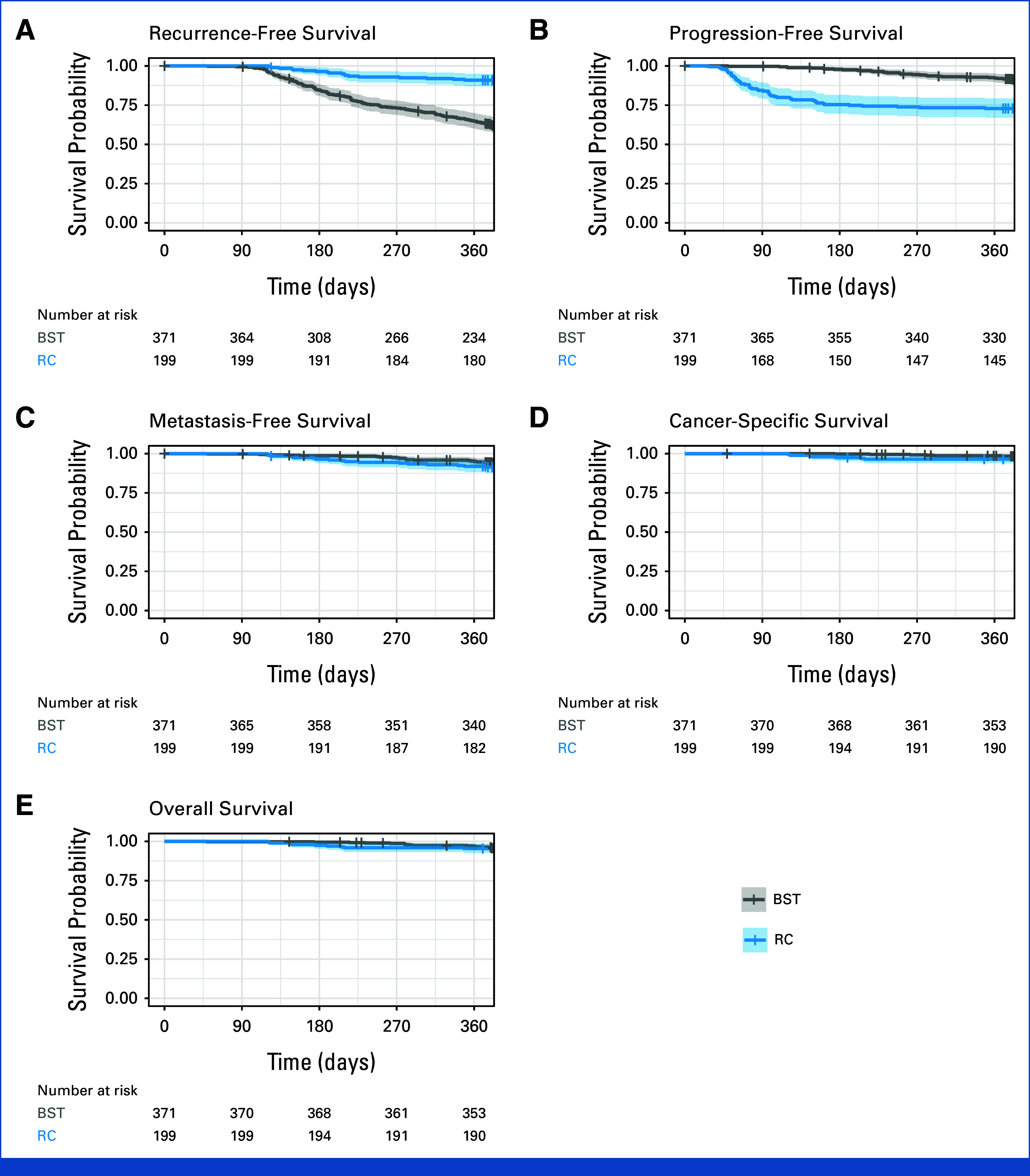
Kaplan-Meier curves for clinical outcomes through 12 months comparing BST and RC for recurrent high-grade non–muscle-invasive bladder cancer. Kaplan-Meier curves for (A) recurrence-free survival, (B) progression-free survival, (C) metastasis-free survival, (D) cancer-specific survival, and (E) overall survival for patients in the BST arm (gray) and RC arm (blue). All clinical outcomes were assessed from the date of the diagnostic transurethral resection of bladder tumor establishing bladder cancer recurrence. Progression-free survival in the RC arm reflects the 22% of patients who had pathology demonstrating MIBC at RC. BST, bladder-sparing therapy; MIBC, muscle-invasive bladder cancer.

Cancer-specific survival did not differ between groups at 12 months (99% for BST *v* 96% for RC; wRR, 0.99 [95% CI, 0.97 to 1.01]; Table 2, Fig 4). Recurrence-free survival was lower for BST (64% *v* 92% for RC; wRR, 1.44 [95% CI, 1.32 to 1.57], Table 2, Fig 4). Within 12 months, 27 (8.1%) participants in the BST arm underwent an eventual RC, including 21 (6.3%) for recurrent high-grade NMIBC and four (1.2%) for progression to MIBC (Data Supplement, Table S12). Because 22% of patients undergoing RC had pathology demonstrating MIBC, 12-month progression-free survival (PFS) was lower for RC (73% *v* 92% for BST; wRR, 0.81 [95% CI, 0.74 to 0.88]; Table 2, Fig 4). Participants in both groups experienced similar metastasis-free survival and OS at 12 months (Table 2, Fig 4).

**TABLE 2.** Clinical Outcomes and SAEs 12 Months After Recurrence of High-Grade Non–Muscle-Invasive Bladder Cancer

Outcome or Event^a^	BST, No./n (%)	RC, No./n (%)	Weighted RR for RC *v* BST (95% CI)^b^
Clinical outcomes			
Cancer recurrence^c^	124/355 (35)	7/187 (3.7)	0.11 (0.05 to 0.24)
Cancer progression^d^	22/350 (6.3)	48/193 (25)	3.84 (2.36 to 6.25)
Metastasis	11/349 (3.2)	5/187 (2.7)	0.82 (0.28 to 2.40)
Bladder cancer–specific mortality^e^	5/365 (1.4)	7/199 (3.5)	2.23 (0.71 to 7.05)
All-cause mortality	13/365 (3.6)	9/199 (4.5)	1.14 (0.49 to 2.66)
Survival outcomes			
Recurrence-free survival^c^	231/361 (64)	180/196 (92)	1.44 (1.32 to 1.57)
PFS^d^	327/357 (92)	145/198 (73)	0.81 (0.74 to 0.88)
Metastasis-free survival	338/357 (95)	182/196 (93)	0.99 (0.96 to 1.03)
Cancer-specific survival^e^	360/365 (99)	192/199 (96)	0.99 (0.97 to 1.01)
OS	352/365 (96)	190/199 (95)	0.99 (0.96 to 1.02)
SAE			
All-cause life-threatening event^f^	7/346 (2.0)	14/190 (7.4)	4.14 (1.63 to 10.47)
Cancer-related life-threatening event^e^^,^^f^	4/346 (1.2)	14/190 (7.4)	7.70 (2.48 to 23.89)
All-cause inpatient hospitalization	47/351 (13)	69/194 (36)	2.71 (1.94 to 3.79)
Cancer-related inpatient hospitalization^e^^,^^g^	29/349 (8.3)	65/194 (34)	4.23 (2.81 to 6.38)

Abbreviations: BST, bladder-sparing therapy; OS, overall survival; PFS, progression-free survival; RC, radical cystectomy; SAEs, serious adverse events.

^a^ Only includes participants with 12 months of follow-up available.

^b^ Inverse probability weighted risk ratios calculated using quasi-Poisson regression and including participants with at least 1 day of follow-up available.

^c^ Recurrence in the urinary tract inclusive of upper urinary tract and urethral recurrences.

^d^ Progression in the RC arm also includes upstaging at the time of RC.

^e^ Includes events of unknown attribution.

^f^ Excludes mortality events.

^g^ Excludes the RC in the RC arm.

### Adverse Events

Participants who selected RC were significantly more likely to experience AEs (62% *v* 38% for BST; wRR, 1.61 [95% CI, 1.35 to 1.91], Data Supplement, Tables S13 and S14), including gastrointestinal, infectious, wound, thromboembolic, and bleeding complications. Participants who selected RC were significantly more likely to experience SAEs and inpatient hospitalizations, both all-cause and cancer-related, compared with those who selected BST (Table 2, Data Supplement, Tables S13 and S14). Among participants who selected RC, 15 (8.0%) experienced prolonged hospitalization after their RC, 57 (30%) were readmitted within 90 days, and five (2.5%) died within 90 days (Data Supplement, Table S12).

## DISCUSSION

In this multicenter, prospective observational cohort study of patients with recurrent high-grade NMIBC, 12-month physical function did not differ between those who chose BST and those who chose RC. Among unpartnered patients and those with concomitant CIS, 12-month physical function was slightly but significantly better after RC, although the differences may have limited clinical relevance. Among patients who contributed to the study design, 66% rated overall QOL as extremely important, behind only survival and the development of metastatic disease.^12^ Furthermore, 76% rated the ability to keep their bladder as very or extremely important, indicative of strong patient preferences against RC. Although our results may seem counterintuitive to conventional wisdom regarding QOL after RC, a recent longitudinal cohort study of 411 patients undergoing RC also demonstrated that physical function and global QOL returns to or exceeds preoperative levels within 6 months.^37^ Thus, for patients with recurrent high-grade NMIBC who are faced with a decision between RC and BST, these results may alleviate concerns about the potential of RC to negatively affect their general QOL compared with BST.

Several secondary outcomes prioritized by patients and relevant to decision making were also assessed.^12,14^ Patients who selected RC reported better mental health and emotional function at 12 months, despite higher anxiety scores at enrollment, possibly reflecting reduced concern about disease recurrence after definitive treatment. These findings suggest that emotional well-being may improve with RC, although forthcoming interview data will further contextualize these results.

Participants in the BST and RC arms endorsed similar financial burden at enrollment. However, by 12 months, participants in the RC arm experienced lower financial difficulties and lower financial toxicity. Although RC is a morbid surgery with a high initial burden, after recovery, those patients mostly experience low health care utilization. Conversely, patients undergoing BST have several induction and maintenance treatments in the year after their recurrence coupled with quarterly surveillance cystoscopies. The financial burden of BST may increase going forward, given recent US Food and Drug Administration approvals of nadofaragene and nogapendekin for BCG-unresponsive NMIBC, which are substantially more expensive than currently available BSTs.

Urinary health was worse at enrollment among participants who selected RC but was similar by 12 months. However, sexual and bowel function remained better in the BST arm throughout follow-up. These findings align with known consequences of RC in men and women that can affect sexual function as well as use of bowel segments in urinary diversion that may alter bowel function.

Upstaging occurred among 22% of patients undergoing RC, reflective of the higher cancer severity in the RC arm and well-established understaging of bladder cancer with imaging and endoscopy.^38^ Despite worse recurrence-free survival in the BST arm and worse PFS in the RC arm, 12-month cancer-specific survival and all-cause mortality did not differ. Postoperative mortality was 3% for patients undergoing RC, with a higher incidence of inpatient hospitalizations compared with patients who selected BST, which is an important tradeoff and underscores the early morbidity of RC. Extended follow-up will provide critical long-term patient-reported and clinical outcomes as these events accrue beyond 12 months.

Although the observational trial design is a limitation of the CISTO study, it was selected on the basis of low patient-reported willingness to be randomly assigned to a trial comparing RC with BST.^12^ Supporting this, a recent feasibility trial randomly assigning patients to RC versus intravesical BCG for newly diagnosed NMIBC did not progress because of low accrual.^13^ Thus, the observational design was the highest-quality feasible study to generate evidence for or against the benefit of RC in recurrent high-grade NMIBC. The trial population was representative of those with bladder cancer in the United States.^39^ Patients were similar between treatment arms with respect to sex, comorbidity burden, education, and employment status. We used modern treatment selection models to balance the observed differences between treatment arms. Because this was a pragmatic trial, the protocol did not specify individual BSTs or RC approach, which allows for the CISTO study to provide generalizable results. Although heterogeneity of treatment strategies within the BST arm may be considered a limitation, results were consistent between ITT and as-treated analyses. This variability reflects contemporary practice, supporting the real-world relevance of our findings.

The findings of the CISTO study support the continued role of RC in recurrent high-grade NMIBC. Although guidelines advocate consideration of RC for patients with recurrent high-grade NMIBC,^4^ current and planned clinical trials for NMIBC do not include RC as a comparator. Thus, to our knowledge, the CISTO study is the only contemporary prospective study evaluating RC as a treatment in recurrent high-grade NMIBC that provides clinical and patient-reported outcomes in order to generate evidence to inform the decision between RC and BST. The CISTO study demonstrates that by 12 months, physical function is similar between patients who retain their bladders and undergo BST and those undergoing RC. Urinary function outcomes 12 months after enrollment were also similar between treatment arms. RC patients reported better mental health and emotional function outcomes and lower financial burden 12 months after enrollment, but worse sexual and bowel function. RC has notable morbidity, with 3% perioperative mortality and 30% of patients experiencing postoperative readmission in the CISTO study consistent with previous trials in RC patients.^40^ However, 12-month cancer-specific survival and all-cause mortality were similar between treatment arms. As patients navigate the challenge of shared decision making in recurrent high-grade NMIBC, these results provide valuable insights into early functional and QOL outcomes. Ongoing collection of these outcomes through 5 years will help clarify the longer-term comparative effectiveness of RC and BST.

## Data Availability

A data sharing statement provided by the authors is available with this article at DOI https://doi.org/10.1200/JCO-25-01324.

## APPENDIX. CISTO COLLABORATIVE MEMBERS

John L. Gore, MD, MS (Department of Urology, University of Washington, Seattle, WA), Angela B. Smith, MD, MS (Department of Urology, School of Medicine, University of North Carolina at Chapel Hill, Chapel Hill, NC), Erika M. Wolff, PhD (Department of Urology, University of Washington, Seattle, WA), Bryan A. Comstock, MS (Department of Biostatistics, University of Washington, Seattle, WA), Michael G. Nash, MS (Department of Biostatistics, University of Washington, Seattle, WA), Kristin M. Follmer, BA (Department of Urology, University of Washington, Seattle, WA), Jenney R. Lee, MA (Department of Urology, University of Washington, Seattle, WA), Sung Min Kim, BS, BA (Department of Urology, University of Washington, Seattle, WA), Larry G. Kessler, ScD (Department of Health Systems and Population Health, School of Public Health, University of Washington, Seattle, WA), Scott M. Gilbert, MD, MS (Department of Genitourinary Oncology, H. Lee Moffit Cancer Center and Research Institute, Tampa, FL), Sam S. Chang, MD, MBA (Department of Urology, Vanderbilt University Medical Center, Nashville, TN), Jonathan L. Wright, MD, MS (Department of Urology, University of Washington, Seattle, WA), Max R. Kates, MD (Department of Urology, The James Brady Urological Institute, Johns Hopkins University School of Medicine, Baltimore, MD), Kamal S. Pohar, MD (Department of Urology, The Ohio State University, Columbus, OH), Thomas J. Guzzo, MD, MPH (Division of Urology, Department of Surgery, University of Pennsylvania Perelman School of Medicine, Philadelphia, PA), Trinity J. Bivalacqua, MD, PhD (Division of Urology, Department of Surgery, University of Pennsylvania Perelman School of Medicine, Philadelphia, PA), Kenneth G. Nepple, MD (Department of Urology, University of Iowa, Iowa City, IA), Jeffrey S. Montgomery, MD, MHSA (Department of Urology, Michigan Medicine, Ann Arbor, MI), Kristen R. Scarpato, MD, MPH (Department of Urology, Vanderbilt University Medical Center, Nashville, TN), Solomon L. Woldu, MD (Department of Urology, University of Texas Southwestern Medical Center, Dallas, TX), Viraj A. Master, MD, PhD (Department of Urology and Winship Cancer Institute, Emory University, Atlanta, GA), David Y.T. Chen, MD (Department of Urology, Fox Chase Cancer Center, Philadelphia, PA), Matthew Mossanen, MD, MPH (Department of Urology, Brigham and Women's Hospital, Boston, MA), Siamak Daneshmand, MD (Department of Urology, Keck School of Medicine, University of Southern California, Los Angeles, CA), Brock B. O'Neil, MD (Division of Urology, Huntsman Cancer Institute, University of Utah, Salt Lake City, UT), Mark D. Tyson, MD, MPH (Department of Urology, Mayo Clinic, Scottsdale, AZ), Mary E. Westerman, MD (Department of Urology, University of North Carolina at Chapel Hill, Chapel Hill, NC), Ashish M. Kamat, MD (Department of Urology, University of Texas MD Anderson Cancer Center, Houston, TX), Ahmed M. Mansour, MD, MRCS (Department of Urology, University of Texas Health, San Antonio, TX), Karim Chamie, MD, MS (Department of Urology, David Geffen School of Medicine at UCLA, Los Angeles, CA), Stephen B. Riggs, MD (Department of Urology, Levine Cancer Institute, Atrium Health, Charlotte, NC), Janet B. Kukreja, MD (Department of Urology, University of Colorado, Denver, CO), Parth K. Modi, MD, MS (Section of Urology, Department of Surgery, University of Chicago, Chicago, IL), Tullika Garg, MD, MPH (Department of Urology, Geisinger Health System, Danville, PA), Charles C. Peyton, MD (Department of Urology, University of Alabama, Birmingham, AL), Jeffrey W. Nix, MD, MSHA (Department of Urology, University of Alabama, Birmingham, AL), Rian Dickstein, MD (Chesapeake Urology, Baltimore, MD), Adam J. Gadzinski, MD, MS (Comprehensive Urology, Royal Oak, MI), Alex Sankin, MD, MS (Department of Urology, Montefiore Medical Center and Albert Einstein School of Medicine, Bronx, NY), Neal D. Shore, MD, FACS (Carolina Urologic Research Center, Myrtle Beach, SC), Brian R. Lane, MD, PhD (Division of Urology, Corewell Health West, Grand Rapids, MI), Jeffrey C. Bassett, MD, MPH (Department of Urologic Oncology, Hoag Memorial Presbyterian Hospital, Newport Beach, CA), Sanjay Patel, MD (Department of Urology, University of Oklahoma Health Sciences Center, Oklahoma City, OK), David S. Morris, MD (Urology Associates, PC, Nashville, TN), Liam C. Macleod, MD, MPH (Department of Urology and Heiman Cancer Center, Asante Rogue Regional Medical, Center, Medford, OR), Eugene K. Lee, MD (Department of Urology, University of Kansas Medical Center, Kansas City, KS), Chad R. Ritch, MD, MBA (Desai Sethi Urology Institute, University of Miami Miller School of Medicine, Miami, FL), Stephanie Chisolm, PhD (Bladder Cancer Advocacy Network, Bethesda, MD), Douglas B. MacLean, MBA (CISTO Advocate Advisory Board, Duvall, WA), Fred Almeida (CISTO Advocate Advisory Board, Roseville, CA), Mary Beth Ballard Murray, MEd (CISTO Advocate Advisory Board, Danville, KY), Nancy Lindsey, BA (CISTO Advocate Advisory Board, Charlotte, NC), Robert Lipman (CISTO Advocate Advisory Board, Bethesda, MD), Richard M. Oliver (CISTO Advocate Advisory Board, Fuquay-Varina, NC), Lori A. Roscoe, PhD (CISTO Advocate Advisory Board, University of South Florida, Tampa, FL), Karen Sachse (CISTO Advocate Advisory Board, Sterling, VA), Yair Lotan, MD (Department of Urology, University of Texas Southwestern Medical Center, Dallas, TX), Sima P. Porten, MD, MPH (Department of Urology, UCSF School of Medicine, San Francisco, CA), Jennifer M. Taylor, MD, MPH (Scott Department of Urology, Baylor College of Medicine, Houston, TX), Gary D. Steinberg, MD (Department of Urology, Rush University Medical Center, Chicago, IL), James W.F. Catto, MD, PhD (Division of Clinical Medicine, School of Medicine & Population Health, University of Sheffield, Sheffield, UK), Tracy M. Downs, MD, FACS (Department of Urology, University of Virginia School of Medicine, Charlottesville, VA), Jennifer L. Malin, MD, PhD (West Los Angeles Veterans Affairs Medical Center, Los Angeles, CA), Elai Davicioni, PhD (Veracyte, Inc, San Diego, CA) Patricia Rios, MPH, MBA (Bladder Cancer Advocacy Network, Bethesda, MD), Marielle Yano, BS (Department of Urology, University of Washington, Seattle, WA), Grace Jun, BS (Department of Urology, University of Washington, Seattle, WA), Casey Li, BS (Department of Urology, University of Washington, Seattle, WA), Christopher Nefcy, BS (Department of Biostatistics, University of Washington, Seattle, WA), Anirban Basu, PhD, MS (Departments of Pharmacy, Health Services, and Economics, University of Washington, Seattle, WA), Patrick J. Heagerty, PhD (Department of Biostatistics, University of Washington, Seattle, WA)

## References

[1] Siegel RL Kratzer TB Giaquinto AN et al Cancer statistics, 2025 CA Cancer J Clin 75 10 45 2025 39817679 10.3322/caac.21871 PMC11745215

[2] Kamat AM Hahn NM Efstathiou JA et al Bladder cancer Lancet (London, England) 388 2796 2810 2016 27345655 10.1016/S0140-6736(16)30512-8

[3] Power NE Izawa J Comparison of guidelines on non-muscle invasive bladder cancer (EAU, CUA, AUA, NCCN, NICE) Bladder Cancer (Amsterdam, the Netherlands) 2 27 36 2016 10.3233/BLC-150034 PMC4927900 27376122

[4] Chang SS Boorjian SA Chou R et al Diagnosis and treatment of non-muscle invasive bladder cancer: AUA/SUO guideline J Urol 196 1021 1029 2016 27317986 10.1016/j.juro.2016.06.049

[5] Botteman MF Pashos CL Redaelli A et al The health economics of bladder cancer: A comprehensive review of the published literature PharmacoEconomics 21 1315 1330 2003 14750899 10.1007/BF03262330

[6] Aly A Johnson C Doleh Y et al The real-world lifetime economic burden of urothelial carcinoma by stage at diagnosis J Clin Pathw 6 51 60 2020 32832698 PMC7433100

[7] Holzbeierlein JM Bixler BR Buckley DI et al Diagnosis and treatment of non-muscle invasive bladder cancer: AUA/SUO guideline: 2024 amendment J Urol 211 533 538 2024 38265030 10.1097/JU.0000000000003846

[8] Kamat AM Sylvester RJ Böhle A et al Definitions, end points, and clinical trial designs for non-muscle-invasive bladder cancer: Recommendations from the International Bladder Cancer Group J Clin Oncol 34 1935 1944 2016 26811532 10.1200/JCO.2015.64.4070 PMC5321095

[9] Sylvester RJ van der Meijden APM Oosterlinck W et al Predicting recurrence and progression in individual patients with stage Ta T1 bladder cancer using EORTC risk tables: A combined analysis of 2596 patients from seven EORTC trials Eur Urol 49 466 477 2006 ; discussion 475 16442208 10.1016/j.eururo.2005.12.031

[10] Flaig TW Spiess PE Abern M et al NCCN guidelines® insights: Bladder cancer, version 3.2024 J Natl Compr Cancer Netw 22 216 225 2024 10.6004/jnccn.2024.0024 38754471

[11] Chou R Buckley D Fu R et al Emerging Approaches to Diagnosis and Treatment of Non–muscle-Invasive Bladder Cancer Rockville, MD Agency for Healthcare Research and Quality (US) 2015 26632629

[12] Smith AB Lee JR Lawrence SO et al Patient and public involvement in the design and conduct of a large, pragmatic observational trial to investigate recurrent, high-risk non-muscle-invasive bladder cancer Cancer 128 103 111 2022 34495550 10.1002/cncr.33897

[13] Catto JWF Gordon K Collinson M et al Radical cystectomy against intravesical BCG for high-risk high-grade nonmuscle invasive bladder cancer: Results from the randomized controlled BRAVO-feasibility study J Clin Oncol 39 202 214 2021 33332191 10.1200/JCO.20.01665 PMC8078404

[14] Gore JL Wolff EM Comstock BA et al Protocol of the comparison of intravesical therapy and surgery as treatment options (CISTO) study: A pragmatic, prospective multicenter observational cohort study of recurrent high-grade non-muscle invasive bladder cancer BMC Cancer 23 1127 2023 10.1186/s12885-023-11605-8 37980511 PMC10657633

[15] Aaronson NK Ahmedzai S Bergman B et al The European Organization for Research and Treatment of Cancer QLQ-C30: A quality-of-life instrument for use in international clinical trials in oncology J Natl Cancer Inst 85 365 376 1993 8433390 10.1093/jnci/85.5.365

[16] Balar AV Kamat AM Kulkarni GS et al Pembrolizumab monotherapy for the treatment of high-risk non-muscle-invasive bladder cancer unresponsive to BCG (KEYNOTE-057): An open-label, single-arm, multicentre, phase 2 study Lancet Oncol 22 919 930 2021 34051177 10.1016/S1470-2045(21)00147-9

[17] Boorjian SA Alemozaffar M Konety BR et al Intravesical nadofaragene firadenovec gene therapy for BCG-unresponsive non-muscle-invasive bladder cancer: A single-arm, open-label, repeat-dose clinical trial Lancet Oncol 22 107 117 2021 33253641 10.1016/S1470-2045(20)30540-4 PMC7988888

[18] Chamie K Chang SS Kramolowsky E et al IL-15 superagonist NAI in BCG-unresponsive non-muscle-invasive bladder cancer NEJM Evid 2 EVIDoa2200167 2023 38320011 10.1056/EVIDoa2200167

[19] Cella D Riley W Stone A et al The Patient-Reported Outcomes Measurement Information System (PROMIS) developed and tested its first wave of adult self-reported health outcome item banks: 2005-2008 J Clin Epidemiol 63 1179 1194 2010 20685078 10.1016/j.jclinepi.2010.04.011 PMC2965562

[20] Herdman M Gudex C Lloyd A et al Development and preliminary testing of the new five-level version of EQ-5D (EQ-5D-5L) Qual Life Res 20 1727 1736 2011 21479777 10.1007/s11136-011-9903-x PMC3220807

[21] de Souza JA Yap BJ Wroblewski K et al Measuring financial toxicity as a clinically relevant patient-reported outcome: The validation of the COmprehensive Score for Financial Toxicity (COST) Cancer 123 476 484 2017 27716900 10.1002/cncr.30369 PMC5298039

[22] Gilbert SM Wood DP Dunn RL et al Measuring health-related quality of life outcomes in bladder cancer patients using the Bladder Cancer Index (BCI) Cancer 109 1756 1762 2007 17366596 10.1002/cncr.22556

[23] U.S. Department of Health and Human Services Common Terminology Criteria for Adverse Events (CTCAE) Version 5.0 https://ctep.cancer.gov/protocoldevelopment/electronic_applications/docs/ctcae_v5_quick_reference_5x7.pdf

[24] Udovicich C Perera M Huq M et al Hospital volume and perioperative outcomes for radical cystectomy: A population study BJU Int 119 26 32 2017 suppl 5 28544301 10.1111/bju.13827

[25] Gore JL Wright JL Daratha KB et al Hospital-level variation in the quality of urologic cancer surgery Cancer 118 987 996 2012 21792864 10.1002/cncr.26373 PMC3273633

[26] R Core Team: R: A Language and Environment for Statistical Computing Vienna, Austria 2025 https://R-project.org/

[27] Rosenblum M van der Laan MJ Targeted maximum likelihood estimation of the parameter of a marginal structural model Int J Biostat 6 Article 19 2010 10.2202/1557-4679.1238 PMC3126671 21731532

[28] Schuler MS Rose S Targeted maximum likelihood estimation for causal inference in observational studies Am J Epidemiol 185 65 73 2017 27941068 10.1093/aje/kww165

[29] Gruber S van der Laan MJ A targeted maximum likelihood estimator of a causal effect on a bounded continuous outcome Int J Biostat 6 Article 26 2010 21731529 10.2202/1557-4679.1260 PMC3126669

[30] Pirracchio R Petersen ML van der Laan M Improving propensity score estimators’ robustness to model misspecification using super learner Am J Epidemiol 181 108 119 2015 25515168 10.1093/aje/kwu253 PMC4351345

[31] Chesnaye NC Stel VS Tripepi G et al An introduction to inverse probability of treatment weighting in observational research Clin Kidney J 15 14 20 2022 35035932 10.1093/ckj/sfab158 PMC8757413

[32] Greenland S Model-based estimation of relative risks and other epidemiologic measures in studies of common outcomes and in case-control studies Am J Epidemiol 160 301 305 2004 15286014 10.1093/aje/kwh221

[33] Lumley T Gao P Schneider B Survey: Analysis of Complex Survey Samples 2024 https://cran.r-project.org/web/packages/survey/index.html

[34] van Buuren S Groothuis-Oudshoorn K Mice: Multivariate imputation by chained equations in R J Stat Softw 45 1 67 2011

[35] US Department of Agriculture, Economic Research Service: 2020 Rural-Urban Commuting Area (RUCA) Codes 2025 https://ers.usda.gov/data-products/rural-urban-commuting-area-codes/documentation

[36] Kind AJH Buckingham WR Making neighborhood-disadvantage metrics accessible—The neighborhood Atlas N Engl J Med 378 2456 2458 2018 29949490 10.1056/NEJMp1802313 PMC6051533

[37] Clements MB Atkinson TM Dalbagni GM et al Health-related quality of life for patients undergoing radical cystectomy: Results of a large prospective cohort Eur Urol 81 294 304 2022 34629182 10.1016/j.eururo.2021.09.018 PMC8891075

[38] Hollenbeck BK Miller DC Dunn RL et al The effects of stage divergence on survival after radical cystectomy for urothelial cancer Urol Oncol 23 77 81 2005 15869990 10.1016/j.urolonc.2004.08.012

[39] Zang Y Li X Cheng Y et al An overview of patients with urothelial bladder cancer over the past two decades: A Surveillance, Epidemiology, and End Results (SEER) study Ann Transl Med 8 1587 2020 33437786 10.21037/atm-20-2108 PMC7791213

[40] Catto JWF Khetrapal P Ricciardi F et al Effect of robot-assisted radical cystectomy with intracorporeal urinary diversion vs open radical cystectomy on 90-Day morbidity and mortality among patients with bladder cancer: A randomized clinical trial JAMA 327 2092 2103 2022 35569079 10.1001/jama.2022.7393 PMC9109000

