# Supplementary material for: Twelve-Month Results From the CISTO Study Comparing Radical Cystectomy Versus Bladder-Sparing Therapy for Recurrent High-Grade Non–Muscle-Invasive Bladder Cancer
Source: J Clin Oncol. 2025 Dec 15;44(4):274–85. doi: 10.1200/JCO-25-01324 (PMC12707586; doi:10.1200/JCO-25-01324)
Supplement: Supplementary file 2 [file jco-44-274-s002.pdf]

Data Supplement

Table of Contents

Methods ... 2

eTable 1. The CISTO Study inclusion and exclusion criteria. ... 2

eTable 2. Categories of recurrent high-grade NMIBC included in the CISTO Study ... 3

eTable 3. Definitions of CISTO Study clinical outcomes at 12-months ... 4

eTable 4. Baseline variables included in TMLE models and propensity score models used to generate inverse probability weights ... 6

eTable 5. Missingness of CISTO Study baseline characteristics and outcomes treatment arm ... 7

eTable 6. Observed CISTO Study data from all participants and tenfold multiple imputation for missing covariates and the primary outcome ... 9

Results ... 10

eTable 7. Representativeness of CISTO Study participants ... 10

eTable 8. All available baseline characteristics of participants in the CISTO Study. ... 11

eTable 9. Actual treatments received by CISTO Study participants in each group ... 15

eFigure 1. Adjusted domain and scale scores 12 months after enrollment for patients in the Bladder-Sparing Therapy (BST) and Radical Cystectomy (RC) arms ... 16

eTable 10. Primary and key secondary patient-reported outcomes at baseline and 12 months by treatment group ... 17

eTable 11. Average treatment effects for each outcome using GEE as a sensitivity analysis ... 18

eTable 12. Treatment arm-specific clinical outcomes 12 months after recurrence of high-grade non-muscle invasive bladder cancer ... 19

eTable 13. Counts of adverse events categorized by grade reported within 12 months of study enrollment by arm ... 20

eTable 14. Proportion of affected participants by arm that experienced an adverse event (AE) or serious adverse event (SAE) by organ system ... 21

References ... 22

Methods

eTable 1. The CISTO Study inclusion and exclusion criteria.

Inclusion Criteria	Exclusion Criteria
1. Adult 18 years of age or older; and<br>2. Presenting with high-grade NMIBC established by anatomic pathology as tumor stage classification Tis, Ta, or T1, and with:<br>a. Pathology documentation from any hospital/clinic/medical center, and<br>b. More than 50% urothelial carcinoma component in the specimen; and<br>3. History of high-grade NMIBC established by anatomic pathology as tumor stage classification Tis, Ta, or T1; and<br>4. Attempted or received induction BCG (at least 3 out of 6 instillations) at any point in time; and<br>5. In the previous 12 months, received at least one instillation of any intravesical agent (induction or maintenance) or one administration of systemic therapy for NMIBC treatment.	1. Any plasmacytoid or small cell (neuroendocrine) component in the pathology (past or current presentation);[a]<br>2. Previous history of cystectomy or radiation therapy for bladder cancer;<br>3. Previous history of muscle-invasive bladder cancer or metastatic bladder cancer;<br>4. Any history of upper tract urothelial carcinoma;<br>5. Incarcerated in a detention facility or in police custody (patients wearing a monitoring device can be enrolled) at baseline/screening;<br>6. Contraindication to radical cystectomy (e.g., ASA classification of 4, cancer does not warrant consideration of cystectomy);<br>7. Contraindication to medical therapy (i.e., intolerant of all medical therapies);<br>8. Unable to provide written informed consent in English;<br>9. Unable to be contacted for research surveys;<br>10. Planning to participate in a Phase I or Phase II interventional clinical trial for NMIBC (unless in the control/comparator arm of a Phase II trial) or any blinded interventional trial for NMIBC.

Abbreviations: NMIBC, non-muscle invasive bladder cancer; BCG, Bacillus Calmette-Guérin; ASA, American Society of Anesthesiologists.

[a]These specific variant histologies were excluded as guidelines advise treatment with radical cystectomy[1]

eTable 2. Categories of recurrent high-grade NMIBC included in the CISTO Study.

Category	Definition
1a. BCG Unresponsive	Persistent or recurrent high-grade NMIBC (stage Tis, Ta, or T1) determined at 6-month evaluation. Patient must have had 5 out of 6 induction BCG instillations and 2 out of 3 maintenance BCG instillations.
1b. BCG Unresponsive[a]	Any stage progression at 3-month evaluation despite 5 out of 6 induction BCG instillations.
2. Relapsing	Recurrent high-grade NMIBC more than 6 months after diagnosis. Patient must have had at least 3 out of 6 induction BCG instillations and had a maintenance BCG instillation within the last 6 months.
3. No recent BCG	Had at least 3 of 6 induction BCG instillations at any point in time. Must have had at least one instillation of some type of intravesical agent (induction or maintenance; postop instillation does not apply) or one administration of systemic therapy approved for NMIBC treatment in last 12 months.

Abbreviations: NMIBC, non-muscle invasive bladder cancer; BCG, Bacillus Calmette-Guérin.
[a]Typically excluded from clinical trials.

eTable 3. Definitions of CISTO Study clinical outcomes at 12-months.

Clinical Outcome	Definition[a]	Exposure time
Cancer recurrence	Any subsequent episode of high-grade bladder cancer in the bladder or elsewhere in the urinary tract (penile urethra, prostatic urethra, ureters, renal pelvis), either NMIBC or MIBC (for cystectomy arm this excludes any finding at cystectomy)	365 days if event occurred, otherwise minimum of 365 days or time from TURBT to last clinical encounter, 90 days following a surveillance procedure with negative findings, or death
Cancer progression	Any subsequent episode of muscle-invasive (T2-T4) disease in the bladder or elsewhere in the urinary tract AND/OR locoregional (true pelvic/common iliac) nodal disease (N+) AND/OR distant metastasis (M+) (for cystectomy arm this includes findings at cystectomy)	365 days if event occurred, otherwise minimum of 365 days or time from TURBT to last clinical encounter, 90 days following a surveillance procedure with negative findings, or death
Metastasis	Metastasis to distant lymph/nodes organs (pathologic or radiologic diagnosis of M+ disease) (for cystectomy arm this includes findings at cystectomy)	365 days if event occurred, otherwise minimum of 365 days or time from TURBT to last clinical encounter, 90 days following a surveillance procedure with negative findings, or death
Bladder cancer-specific mortality	Death due to cancer or treatment	365 days if event occurred, otherwise minimum of 365 days or time from TURBT to last date on which the participant's vital status could be verified
All-cause mortality	Death due any cause	365 days if event occurred, otherwise minimum of 365 days or time from TURBT to last date on which the participant's vital status could be verified
Recurrence-free survival	No event (recurrence or death)	365 days if event occurred, otherwise minimum of 365 days or time from TURBT to last clinical encounter, 90 days following a surveillance procedure with negative findings, or death
Progression-free survival	No event (progression or metastasis or death)	365 days if event occurred, otherwise minimum of 365 days or time from TURBT to last clinical encounter, 90 days following a surveillance procedure with negative findings, or death
Metastasis-free survival	No event (metastasis or death)	365 days if event occurred, otherwise minimum of 365 days or time from TURBT to last clinical encounter, 90 days following a surveillance procedure with negative findings, or death
Cancer-specific survival	No event (cancer-related death)	365 days if event occurred, otherwise minimum of 365 days or time from TURBT to last date on which the participant's vital status could be verified or death unrelated to cancer
Overall survival	No event (death from any cause)	365 days if event occurred, otherwise minimum of 365 days or time from TURBT to last date on which the participant's vital status could be verified or death from any cause
All-cause life-threatening event	SAE including life-threatening event due to any cause	365 days if event occurred, otherwise minimum of 365 days or time from TURBT to last date on which the participant's vital status could be verified
Cancer-related life-threatening event	SAE including life-threatening event due to cancer or treatment	365 days if event occurred, otherwise minimum of 365 days or time from TURBT to last date on which the participant's vital status could be verified
All-cause inpatient hospitalization	SAE including hospitalization due to any cause	365 days if event occurred, otherwise minimum of 365 days or time from TURBT to last date on which the participant's vital status could be verified

Cancer-related inpatient hospitalization	SAE including hospitalization due to cancer or treatment	365 days if event occurred, otherwise minimum of 365 days or time from TURBT to last date on which the participant's vital status could be verified

[a]All clinical outcomes were assessed within 365 days of the diagnostic TURBT establishing recurrence of high-grade NMIBC

Abbreviations: TURBT, transurethral resection of bladder tumor; NMIBC, non-muscle invasive bladder cancer; SAE, serious adverse event

eTable 4. Baseline variables included in TMLE models and propensity score models used to generate inverse probability weights.

Variable	Variable Specification
Age	Age at enrollment as continuous and categorical variable
Gender	Male, female
Race/ethnicity	Non-Hispanic White vs. all other racial/ethnic groupings
Comorbid health conditions	Modified Elixhauser Comorbidity Index; PROMIS Emotional Distress-Anxiety/Depression – Short Forms 4a
Smoking history	Active, former, never
Cancer severity	
Stage	Tumor stage classification (T1 or Ta/CIS only, CIS or no CIS)
Cancer histology	Urothelial vs. mixed histology with atypical variant
Partner status	Partnered vs. unpartnered
Social determinants of health	Area Deprivation Index based on ZIP Code;[2] Employment Status; Health Care / Prescription Drug Coverage
Rurality	Urban or rural, based on RUCA code derived from ZIP code[3]
Patient expectations	"What do you believe are the chances that your cancer will go away and never come back with the treatment you have selected?" (greater than 50% or not)
Baseline QOL	Baseline EORTC QLQ-C30 Physical Functioning, BCI Bladder, Urinary, and Sexual Summary, EQ-5D
Baseline outcome	When modeling each outcome, we included the baseline value of that outcome.

Abbreviations: TMLE, targeted maximum likelihood estimation; QOL, quality of life; PROMIS, Patient Reported Outcome Measurement Information System; CIS, *carcinoma in situ*; RUCA, Rural-Urban Commuting Area; EORTC QLQ-C30, European Organisation for Research and Treatment of Cancer Core Quality of Life; BCI, Bladder Cancer Index; EQ-5D: EuroQoL EQ-5D-5L.

eTable 5. Missingness of CISTO Study baseline characteristics and outcomes treatment arm.

Variable, n (%)	Bladder-Sparing Therapy (BST)	Radical Cystectomy (RC)
**Patient characteristics**	**371**	**199**
Age	0 (0.0%)	0 (0.0%)
Gender	0 (0.0%)	0 (0.0%)
Race/ethnicity	0 (0.0%)	0 (0.0%)
Spouse/partner status	4 (1.1%)	1 (0.5%)
Employment status	0 (0.0%)	0 (0.0%)
Health insurance	1 (0.3%)	1 (0.5%)
Rurality	0 (0.0%)	0 (0.0%)
National Area Deprivation Index	0 (0.0%)	0 (0.0%)
Modified Elixihauser comorbidity index	0 (0.0%)	1 (0.5%)
Smoking history	0 (0.0%)	0 (0.0%)
**Tumor characteristics**		
Carcinoma in situ	1 (0.3%)	3 (1.5%)
Clinical T stage T1	0 (0.0%)	0 (0.0%)
Secondary variant histology	1 (0.3%)	2 (1.0%)
**Patient-reported outcomes at enrollment**		
EORTC Physical Functioning	0 (0.0%)	0 (0.0%)
EORTC Global Health	3 (0.8%)	1 (0.5%)
EORTC Role Functioning	3 (0.8%)	0 (0.0%)
EORTC Emotional Functioning	2 (0.5%)	1 (0.5%)
EORTC Cognitive Functioning	2 (0.5%)	1 (0.5%)
EORTC Social Functioning	2 (0.5%)	1 (0.5%)
PROMIS Depression	4 (1.1%)	1 (0.5%)
PROMIS Anxiety	4 (1.1%)	1 (0.5%)
EQ-5D	18 (4.9%)	15 (7.5%)
COST	8 (2.2%)	3 (1.5%)
EORTC Financial Difficulties	4 (1.1%)	2 (1.0%)
BCI Urinary Summary	3 (0.8%)	2 (1.0%)
BCI Urinary Function	16 (4.3%)	8 (4.0%)
BCI Urinary Bother	7 (1.9%)	4 (2.0%)
BCI Bowel Summary	6 (1.6%)	0 (0.0%)
BCI Bowel Function	16 (4.3%)	5 (2.5%)
BCI Bowel Bother	6 (1.6%)	0 (0.0%)
BCI Sexual Summary	51 (14%)	19 (9.5%)
BCI Sexual Function	43 (12%)	18 (9.0%)
BCI Sexual Bother	50 (13%)	20 (10%)
EORTC Fatigue	1 (0.3%)	1 (0.5%)
EORTC Nausea	1 (0.3%)	1 (0.5%)
EORTC Pain	1 (0.3%)	0 (0.0%)
EORTC Dyspnoea	4 (1.1%)	1 (0.5%)
EORTC Insomnia	1 (0.3%)	1 (0.5%)
EORTC Appetite	2 (0.5%)	1 (0.5%)
EORTC Constipation	4 (1.1%)	2 (1.0%)
EORTC Diarrhoea	4 (1.1%)	3 (1.5%)
**Patient-reported outcomes at 12 months[a]**	**344**	**187**
EORTC Physical Functioning	40 (12%)	13 (7.0%)
EORTC Global Health	47 (14%)	16 (8.6%)
EORTC Role Functioning	46 (13%)	16 (8.6%)
EORTC Emotional Functioning	47 (14%)	16 (8.6%)
EORTC Cognitive Functioning	47 (14%)	16 (8.6%)
EORTC Social Functioning	47 (14%)	16 (8.6%)
PROMIS Depression	50 (15%)	18 (9.6%)
PROMIS Anxiety	50 (15%)	18 (9.6%)
EQ-5D	66 (19%)	25 (13%)

Variable, n (%)	Bladder-Sparing Therapy (BST)	Radical Cystectomy (RC)
COST	56 (16%)	20 (11%)
EORTC Financial Difficulties	48 (14%)	18 (9.6%)
BCI Urinary Summary	52 (15%)	21 (11%)
BCI Urinary Function	63 (18%)	38 (20%)
BCI Urinary Bother	56 (16%)	21 (11%)
BCI Bowel Summary	51 (15%)	21 (11%)
BCI Bowel Function	59 (17%)	29 (16%)
BCI Bowel Bother	51 (15%)	25 (13%)
BCI Sexual Summary	106 (31%)	44 (24%)
BCI Sexual Function	102 (30%)	43 (23%)
BCI Sexual Bother	102 (30%)	44 (24%)
EORTC Fatigue	48 (14%)	17 (9.1%)
EORTC Nausea	48 (14%)	16 (8.6%)
EORTC Pain	46 (13%)	16 (8.6%)
EORTC Dyspnoea	47 (14%)	17 (9.1%)
EORTC Insomnia	49 (14%)	16 (8.6%)
EORTC Appetite	49 (14%)	16 (8.6%)
EORTC Constipation	48 (14%)	17 (9.1%)
EORTC Diarrhoea	47 (14%)	16 (8.6%)

[a]All participants who had not withdrawn or passed away during the 12-month survey window.
Abbreviations: EORTC, European Organisation for Research and Treatment of Cancer Core QLQ-C30; PROMIS, Patient Reported Outcome Measurement Information System; EQ-5D: EuroQoL EQ-5D-5L; COST: Comprehensive Score for Financial Toxicity; BCI, Bladder Cancer Index.

eTable 6. Observed CISTO Study data from all participants and tenfold multiple imputation for missing covariates and the primary outcome.

Variable	Observed study data (n=570)	Imputed datasets (m=10)[a]
**Patient characteristics, n (%)**		
Chose cystectomy	199 (35%)	1,990 (35%)
Age ≥ 75	212 (37%)	2,120 (37%)
Female gender	117 (21%)	1,170 (21%)
Non-Hispanic White race/ethnicity	514 (90%)	5,140 (90%)
Has a spouse or partner	458 (81%)	4,620 (81%)
Employed	152 (27%)	1,520 (27%)
Retired	366 (64%)	3,660 (64%)
Private health insurance	195 (34%)	1,954 (34%)
Medicare	403 (71%)	4,044 (71%)
Urban dweller	479 (84%)	4,790 (84%)
National Area Deprivation Index, mean (SD)	41.0 (25.6)	41.0 (25.6)
Modified Elixhauser Comorbidity Index, mean (SD)	1.3 (4.8)	1.3 (4.8)
Ever smoker	373 (65%)	3,730 (65%)
Current smoker	38 (6.7%)	380 (6.7%)
**Tumor characteristics, n (%)**		
Carcinoma in situ	273 (48%)	2,749 (48%)
Clinical T stage T1	223 (39%)	2,230 (39%)
Secondary variant histology	21 (3.7%)	214 (3.8%)
**Patient-reported outcomes at enrollment, mean (SD)**		
EORTC physical functioning	89.1 (16.1)	89.1 (16.1)
PROMIS Depression Short Form 4a	48.4 (8.1)	48.6 (8.5)
PROMIS Anxiety Short Form 4a	52.5 (9.2)	50.3 (9.1)
EuroQoL 5D	0.8 (0.1)	0.8 (0.1)
BCI urinary summary	82.4 (17.4)	82.4 (17.4)
BCI bowel summary	80.1 (9.3)	80.1 (9.3)
BCI sexual summary	48.9 (25.9)	48.5 (25.7)
**Primary outcome at 12 months, mean (SD)**		
EORTC physical functioning	85.5 (18.4)	83.8 (19.7)

[a]Counts in the imputed column are totals summed across the 10 completed datasets. Fully observed baseline variables were specified as predictor-only and not imputed.

Abbreviations: SD, standard deviation; EORTC, European Organisation for Research and Treatment of Cancer Core QLQ-C30; PROMIS, Patient Reported Outcome Measurement Information System; EQ-5D: EuroQoL EQ-5D-5L; BCI, Bladder Cancer Index.

Results

eTable 7. Representativeness of CISTO Study participants.

Category	Considerations
Disease, problem, or condition under investigation	Adult patients with recurrent high-grade NMIBC who are selecting between Bladder-Sparing Therapy and Radical Cystectomy
Special considerations related to	
Sex and gender	In the US, 24% of patients with bladder cancer are female[4]
Age	The incidence of bladder cancer increases with age. Bladder cancer is most frequently diagnosed among people aged 65-74. The average age of bladder cancer diagnosis is 71.[4]
Race or ethnic group	Data from SEER reports that 90% of bladder cancer patients in the US are White, 6% are Black, 4% are Asian or Pacific Islander, and 0.33% are American Indian/Alaskan Native.[5,6]
Geography	Bladder cancer is the ninth most common cancer worldwide. The age-standardized rates of bladder cancer are highest in Southern Europe, Northern Europe, Western Europe, Northern America, and Northern Africa.[7]
Overall representativeness of this trial	The participants in the CISTO Study demonstrated the expected ratio of males to females. In addition, the mean age in the study population was consistent with population-based data from the US. Our trial had similar proportions of White and Black participants compared to US population-based data. Hispanic patients were slightly underrepresented in our trial compared to US population-based data.

Abbreviations: NMIBC, non-muscle invasive bladder cancer; SEER, Surveillance, Epidemiology, and End Results.

eTable 8. All available baseline characteristics of participants in the CISTO Study.

Characteristic	Bladder-Sparing Therapy (BST)	Radical Cystectomy (RC)	Missing N	P value
**Patient characteristics, n (%)**	371	199		
Age (years), mean (SD)	72.4 (8.3)	69.6 (9.1)	0	< .001
≥ 75	150 (40%)	62 (31%)		0.03
Female gender	78 (21%)	39 (20%)	0	0.74
Race/Ethnicity			0	0.10
Non-Hispanic White	332 (89%)	182 (91%)		
Non-Hispanic Black	18 (4.9%)	8 (4.0%)		
Non-Hispanic Asian	6 (1.6%)	4 (2.0%)		
Non-Hispanic American Indian / Alaskan Native	1 (0.3%)	0 (0.0%)		
Non-Hispanic Pacific Islander / Native Hawaiian	0 (0.0%)	0 (0.0%)		
Hispanic	14 (3.8%)	2 (1.0%)		
Multiple	0 (0.0%)	1 (0.5%)		
Other	0 (0.0%)	2 (1.0%)		
Education			13	0.51
Less than High School	20 (5.6%)	4 (2.0%)		
High school graduate or GED	62 (17%)	32 (16%)		
Some college, no degree	76 (21%)	43 (22%)		
Occupational/technical/vocational program	23 (6.4%)	15 (7.6%)		
Associate degree: academic program	25 (6.9%)	14 (7.1%)		
Bachelor's degree	71 (20%)	51 (26%)		
Master's degree	53 (15%)	26 (13%)		
Professional school degree	22 (6.1%)	9 (4.6%)		
Doctoral degree	8 (2.2%)	3 (1.5%)		
Marital status			12	0.009
Married	258 (71%)	152 (77%)		
Divorced	52 (14%)	20 (10%)		
Widowed	41 (11%)	11 (5.6%)		
Separated	2 (0.6%)	5 (2.5%)		
Never married	8 (2.2%)	9 (4.6%)		
Has a partner	265 (75%)	158 (83%)	28	0.06
Has a spouse or partner	288 (78%)	170 (86%)	5	0.03
Has someone who helps with health needs	202 (57%)	112 (59%)	22	0.65
If yes, who most helps with health needs			23	0.21
Spouse	140 (70%)	88 (79%)		
Parent	1 (0.5%)	2 (1.8%)		
Adult child	36 (18%)	10 (8.9%)		
Friend	9 (4.5%)	3 (2.7%)		
Sibling	7 (3.5%)	4 (3.6%)		
Other	8 (4.0%)	5 (4.5%)		
More than one person helps with health needs	68 (19%)	40 (21%)	26	0.65
Employment status			11	0.57
Employed, full time	76 (21%)	37 (19%)		
Employed, part-time	19 (5.2%)	14 (7.1%)		
Employed, but on sick leave or maternity leave	5 (1.4%)	1 (0.5%)		
Unemployed, looking for work	4 (1.1%)	0 (0.0%)		
Unemployed, NOT looking for work	4 (1.1%)	3 (1.5%)		
Disabled, permanently or temporarily	9 (2.5%)	8 (4.1%)		
Retired	238 (66%)	128 (65%)		
Other	7 (1.9%)	6 (3.0%)		
Number living in house, mean (SD)	2.1 (1.0)	2.2 (0.8)	13	0.53
1	63 (18%)	22 (11%)		
2	247 (69%)	142 (72%)		
> 2	50 (14%)	33 (17%)		
Rurality[a]			0	0.06
Urban	323 (87%)	156 (78%)		
Large Rural City/Town	31 (8.4%)	28 (14%)		

Characteristic	Bladder-Sparing Therapy (BST)	Radical Cystectomy (RC)	Missing N	P value
Small Rural Town	8 (2.2%)	7 (3.5%)		
Isolated Small Rural Town	9 (2.4%)	8 (4.0%)		
National Area Deprivation Index,[b] median (IQR)	37.0 (21.0, 61.0)	38.0 (19.5, 61.5)	0	0.95
Total combined household income			25	0.16
Less than $5,000	7 (2.0%)	5 (2.6%)		
$5,000 through $11,999	9 (2.5%)	4 (2.1%)		
$12,000 through $15,999	10 (2.8%)	2 (1.0%)		
$16,000 through $24,999	14 (4.0%)	20 (10%)		
$25,000 through $34,999	16 (4.5%)	10 (5.2%)		
$35,000 through $49,999	33 (9.3%)	19 (9.9%)		
$50,000 through $74,999	57 (16%)	34 (18%)		
$75,000 through $99,999	48 (14%)	19 (9.9%)		
$100,000 and greater	92 (26%)	49 (26%)		
Prefer not to answer	68 (19%)	29 (15%)		
Health insurance			2	
Uninsured	1 (0.3%)	0 (0.0%)		1.00
Private	119 (32%)	76 (38%)		0.14
Medicare	267 (72%)	136 (69%)		0.38
Medicare and private	48 (13%)	28 (14%)		0.70
Medicaid / Medi-Cal	18 (4.9%)	6 (3.0%)		0.38
Other Government (TriCare, VA, IHS)	16 (4.3%)	8 (4.0%)		1.00
Labor and Industry	1 (0.3%)	1 (0.5%)		1.00
Self	1 (0.3%)	0 (0.0%)		1.00
Other / Unknown	25 (6.8%)	14 (7.1%)		0.86
Prescription coverage	347 (96%)	187 (96%)	12	1.00
Ever smoker	247 (67%)	126 (63%)	0	0.46
Current smoker	28 (7.5%)	10 (5.0%)	0	0.29
Pack years smoked, median (IQR)	18.0 (7.8, 38.2)	15.5 (5.5, 27.1)	21	0.01
Body mass index, mean (SD)	29.1 (5.8)	29.2 (5.3)	0	0.83
Body mass index > 25	274 (74%)	155 (78%)	0	0.31
ECOG performance status, mean (SD)	0.2 (0.5)	0.2 (0.4)	41	0.19
Charlson Comorbidity Index (classic), median (IQR)	1.0 (0.0, 2.0)	1.0 (0.0, 2.0)	59	0.90
0	152 (45%)	80 (46%)		
1	65 (19%)	26 (15%)		
2	51 (15%)	34 (20%)		
>2	70 (21%)	33 (19%)		
Charlson Comorbidity Index (updated), median (IQR)	0.0 (0.0, 1.8)	0.0 (0.0, 2.0)	59	0.94
0	216 (64%)	111 (64%)		
1	37 (11%)	13 (7.5%)		
2	55 (16%)	36 (21%)		
>2	30 (8.9%)	13 (7.5%)		
Elixhauser Comorbidity Index, median (IQR)	0.0 (-3.0, 4.0)	0.0 (-3.0, 4.0)	14	0.91
< 0	115 (31%)	60 (32%)		
0	125 (34%)	61 (32%)		
> 0	128 (35%)	67 (36%)		
Modified Elixhauser Comorbidity Index, median (IQR)	0.0 (-1.0, 4.0)	0.0 (-2.0, 4.0)	1	0.89
< 0	96 (26%)	53 (27%)		
0	138 (37%)	71 (36%)		
> 0	137 (37%)	74 (37%)		
Comorbidities				
Blood loss anaemia[d]	6 (1.6%)	5 (2.5%)	0	0.53
Cardiac arrhythmias[d]	58 (16%)	36 (18%)	0	0.48
Cerebrovascular disease[c]	18 (4.9%)	9 (4.5%)	0	1.00
Chronic pulmonary disease[e]	12 (3.2%)	8 (4.0%)	0	0.64
Coagulopathy[d]	10 (2.7%)	4 (2.0%)	0	0.78
Congestive heart failure[e]	17 (4.6%)	3 (1.5%)	0	0.06
Deficiency anaemia[d]	18 (4.9%)	12 (6.0%)	0	0.56

Characteristic	Bladder-Sparing Therapy (BST)	Radical Cystectomy (RC)	Missing N	P value
Depression[d]	35 (9.5%)	18 (9.5%)	13	1.00
Dementia[c]	1 (0.3%)	1 (0.5%)	0	1.00
Chronic pulmonary disease[e]	12 (3.2%)	8 (4.0%)	0	0.64
Diabetes[c]	86 (23%)	35 (18%)	0	0.13
Diabetes with chronic complication[c]	4 (1.2%)	0 (0.0%)	46	0.30
Drug abuse[d]	6 (1.6%)	4 (2.1%)	13	0.74
Fluid and electrolyte disorders[d]	6 (1.6%)	1 (0.5%)	0	0.43
Hemiplegia/paraplegia/paralysis[e]	1 (0.3%)	1 (0.5%)	0	1.00
HIV/AIDS[c]	0 (0.0%)	2 (1.1%)	13	0.11
Leukemia[c]	3 (0.8%)	1 (0.5%)	0	1.00
Lymphoma[e]	4 (1.1%)	1 (0.5%)	0	0.66
Liver disease (any)[e]	5 (1.3%)	4 (2.0%)	0	0.73
Liver disease (moderate or severe)[c]	3 (0.8%)	2 (1.0%)	0	1.00
Any malignancy[e]	64 (17%)	35 (18%)	0	0.91
Metastatic solid tumor[e]	6 (1.6%)	2 (1.0%)	0	0.72
Myocardial infarction[c]	35 (9.4%)	18 (9.0%)	0	1.00
Obesity**	136 (37%)	74 (37%)	0	0.93
Other neurological disorders[d]	1 (0.3%)	1 (0.5%)	0	1.00
Peptic ulcer disease[c]	51 (14%)	28 (14%)	0	0.90
Peripheral vascular disease[e]	21 (5.7%)	13 (6.5%)	0	0.71
Pulmonary circulation disorders[d]	13 (3.5%)	1 (0.5%)	1	0.04
Rheumatoid arthritis/collagen vascular diseases[c]	31 (8.4%)	7 (3.5%)	0	0.03
Renal disease[c]	32 (8.6%)	15 (7.5%)	0	0.75
Renal failure[d]	4 (1.1%)	0 (0.0%)	0	0.30
Valvular disease[d]	26 (7.0%)	3 (1.5%)	0	0.004
Weight loss[d]	2 (0.5%)	3 (1.5%)	0	0.35
**Tumor characteristics, n (%)**				
Carcinoma in situ	165 (45%)	108 (55%)	4	0.08
Clinical T stage			0	< .001
CIS only	108 (29%)	42 (21%)		
Ta	153 (41%)	44 (22%)		
T1	110 (30%)	113 (57%)		
Recurrence category			0	0.08
BCG unresponsive	179 (48%)	88 (44%)		
Relapsing	115 (31%)	53 (27%)		
No recent BCG	77 (21%)	58 (29%)		
Muscularis propria present	203 (58%)	114 (60%)	28	0.58
Secondary variant histology	8 (2.2%)	13 (6.6%)	3	0.01
Adenocarcinoma	0 (0.0%)	0 (0.0%)		
Micropapillary	3 (38%)	3 (23%)		0.63
Sarcomatoid	0 (0.0%)	0 (0.0%)		
Signet ring cell	0 (0.0%)	0 (0.0%)		
Squamous cell	3 (38%)	7 (54%)		0.66
Other	2 (25%)	3 (23%)		1.00
Unknown	0 (0.0%)	0 (0.0%)		
Pathological T stage at radical cystectomy[f]			0	
T0		31 (16%)		
Ta		24 (12%)		
Tis		62 (31%)		
T1		37 (19%)		
T2		24 (12%)		
T3		18 (9.0%)		
T4a		3 (1.5%)		
**Bladder Cancer History**				
Years since initial diagnosis, median (IQR)	1.6 (0.9, 2.9)	1.5 (0.8, 3.1)	0	0.57
<1	112 (30%)	73 (37%)		
1-5	219 (59%)	102 (51%)		

Characteristic	Bladder-Sparing Therapy (BST)	Radical Cystectomy (RC)	Missing N	P value
5+	40 (11%)	24 (12%)		
Instillations of BCG, median (IQR)	9.0 (6.0, 13.0)	9.5 (6.0, 12.0)	5	0.86
<= 6	132 (36%)	76 (38%)		
7-9	60 (16%)	23 (12%)		
10-12	82 (22%)	50 (25%)		
>12	93 (25%)	49 (25%)		
History of non-BCG BST, n (%)[g]	125 (35%)	87 (45%)		0.02
Gemcitabine/Docetaxel	35 (28%)	20 (23%)		0.43
Gemcitabine	28 (22%)	15 (17%)		0.39
Valrubicin	5 (4.0%)	5 (5.7%)		0.74
Mitomycin C	15 (12%)	10 (11%)		1.00
Pembrolizumab	22 (18%)	25 (29%)		0.06
Other	26 (21%)	17 (20%)		0.93
**Patient-reported outcomes at enrollment, mean (SD)**				
EORTC physical functioning[h]	88.6 (16.9)	89.8 (14.5)	0	0.37
PROMIS Depression[i]	48.1 (8.2)	49.0 (8.0)	5	0.21
PROMIS Anxiety[i]	51.7 (9.3)	54.0 (8.8)	5	0.005
EQ-5D[h]	0.84 (0.13)	0.84 (0.12)	33	0.61
BCI urinary summary[h]	84.9 (15.5)	77.7 (19.7)	5	< 0.001
BCI bowel summary[h]	80.9 (9.2)	78.7 (9.4)	6	0.008
BCI sexual summary[h]	49.3 (25.5)	48.2 (26.6)	70	0.66

Abbreviations: SD, standard deviation; GED, General Educational Development; IQR, interquartile range; VA, Veterans Affairs; IHS, Indian Health Service; CIS, carcinoma in situ; BCG, Bacillus Calmette-Guérin, EORTC, European Organisation for Research and Treatment of Cancer Core QLQ-C30; PROMIS, Patient Reported Outcome Measurement Information System; EQ-5D: EuroQoL EQ-5D-5L; BCI, Bladder Cancer Index.

[a]Based on ZIP code and Rural-Urban Commuting Area (RUCA) codes[8]

[b]Based on ZIP code and the 2022 National Area Deprivation Index v4.0[2]

[c]Included in Charlson Comorbidity Index

[d]Included in Elixhauser Comorbidity Index

[e]Included in both Charlson and Elixhauser comorbidity indices

[f]Stage from the radical cystectomy specimen for patients in the radical cystectomy treatment arm.

[g]'Other BST' means BST other than BCG. Proportions of participants having each type of other BST are out of all participants who had other BST in each arm. Types of BST are not mutually exclusive, so percentages will not add to 100%. We did not collect data about individual courses of BST before enrollment and therefore cannot confirm whether drugs were administered as part of the same course or different courses. 'Gemcitabine/Docetaxel' means both gemcitabine and docetaxel were used at some point before study entry TURBT, with or without other drugs. 'Gemcitabine' means gemcitabine was used and docetaxel and pembrolizumab were not. 'Valrubicin', 'Mitomycin C', and 'Pembrolizumab' respectively indicate each drug was used with or without other drugs. 'Other' includes cabazitaxel, CG0070, cisplatin, docetaxel without gemcitabine, durvalumab, EG-70, enfortumab vedotin, erdafitinib, inodifagene vixteplasmid, instiladrin, interferon, N-803, nivolumab, and proleukin.

[h]Higher score means better functioning / health / quality of life

[i]Higher score means worse symptoms

eTable 9. Actual treatments received by CISTO Study participants in each group.

Treatment	N (%)
Cystectomy received in cystectomy arm	198 (99%)
Approach	
Robotic	98 (51%)
Open	91 (47%)
Conversion from robotic to open	3 (1.6%)
Diversion	
Ileal conduit	156 (79%)
Neobladder	37 (19%)
Continent reservoir	4 (2.0%)
Bladder-sparing therapy initiated in BST arm	361 (97%)
Type	
BCG	101 (28%)
BCG combinations[a]	5 (1%)
Gemcitabine/docetaxel	186 (52%)
Gemcitabine	14 (4%)
Valrubicin	2 (1%)
Mitomycin C	4 (1%)
Other intravesical agents[b]	22 (6%)
Pembrolizumab[c]	27 (7%)

Abbreviations: BCG, Bacillus Calmette-Guérin.
[a]Includes BCG + interferon (2), BCG + Anktiva (2), BCG + rapamycin (1)
[b]Includes CG0070 (12), Adstiladrin (4), valrubicin/docetaxel (3), detalimogene voraplasmid (1), paclitaxel (1), TAR-200 (1)
[c]Includes pembrolizumab/gemcitabine (8), pembrolizumab/gemcitabine/cabazitaxel (1)

eFigure 1. Adjusted domain and scale scores 12 months after enrollment for patients in the Bladder-Sparing Therapy (BST) and Radical Cystectomy (RC) arms.

	Bladder-Sparing Therapy	Radical Cystectomy	Average Treatment Effect (95% CI)
**EORTC Physical Functioning Subgroups**			
Males (N=240, 138)	84.4 (19.6)	87.1 (17.3)	1.0 (-0.7, 2.7)
Females (N=64, 36)	87.0 (16.0)	84.1 (18.3)	0.2 (-2.4, 2.9)
75 or older (N=123, 55)	82.0 (20.2)	83.9 (17.4)	-0.4 (-2.3, 1.4)
Under 75 (N=181, 119)	87.0 (17.7)	87.7 (17.4)	1.3 (-0.7, 3.2)
Has partner (N=240, 151)	86.0 (18.9)	87.3 (17.2)	0.7 (-1.0, 2.5)
No partner (N=61, 22)	80.6 (18.7)	82.8 (17.4)	3.3 (0.1, 6.4)
Carcinoma in situ (N=138, 92)	83.3 (19.8)	86.5 (16.3)	2.7 (0.8, 4.7)
No carcinoma in situ (N=166, 79)	86.4 (18.0)	86.6 (18.9)	-0.3 (-2.2, 1.7)
**Bladder Cancer Index**			
Urinary Function (N=281, 149)	80.2 (26.2)	80.5 (26.0)	4.0 (1.5, 6.5)
Urinary Bother (N=288, 166)	88.0 (15.7)	85.3 (14.1)	-0.1 (-2.0, 1.9)
Bowel Function (N=285, 158)	84.1 (15.4)	80.7 (18.5)	-1.5 (-3.1, 0.2)
Bowel Bother (N=293, 162)	77.4 (9.7)	75.2 (10.6)	-1.0 (-2.2, 0.1)
Sexual Function (N=242, 144)	34.9 (28.7)	21.2 (22.4)	-12.4 (-16.1, -8.8)
Sexual Bother (N=242, 143)	64.6 (29.3)	48.8 (25.6)	-10.0 (-13.3, -6.6)
**EORTC Symptom Scales**			
Fatigue (N=296, 170)	25.8 (22.3)	26.8 (20.6)	-1.0 (-3.3, 1.4)
Nausea (N=296, 171)	3.7 (10.5)	3.1 (8.9)	-1.4 (-2.7, -0.2)
Pain (N=298, 171)	17.0 (24.2)	16.1 (24.8)	-2.5 (-4.8, -0.3)
Dyspnoea (N=297, 170)	16.2 (24.1)	12.9 (19.6)	-3.0 (-5.8, -0.2)
Insomnia (N=295, 171)	26.3 (26.7)	26.3 (26.4)	-2.7 (-5.3, -0.2)
Appetite (N=295, 171)	11.2 (20.7)	9.9 (20.4)	-3.9 (-6.1, -1.6)
Constipation (N=296, 170)	15.1 (23.1)	18.4 (24.6)	1.8 (-1.3, 5.0)
Diarrhoea (N=297, 171)	8.4 (16.4)	11.9 (20.0)	2.2 (0.2, 4.2)

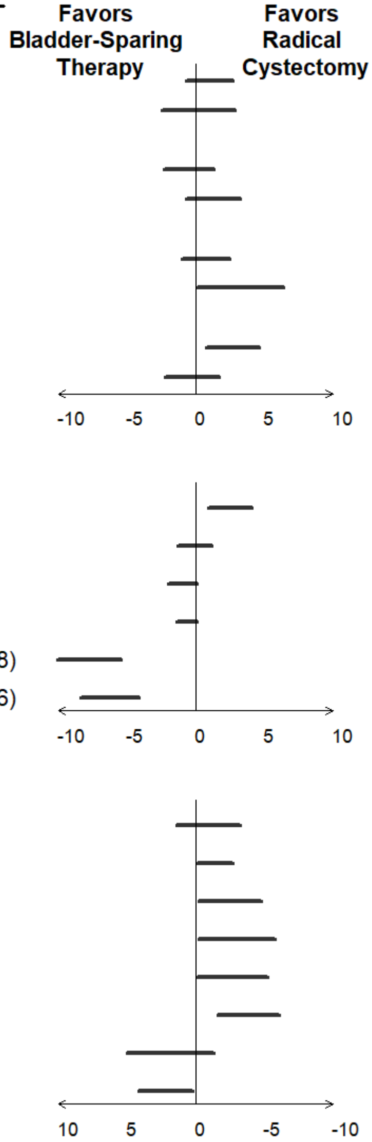

Unadjusted domain and scale mean scores and standard deviations at 12 months after enrollment are shown for patients in the BST and RC arms who completed each measure. Adjusted average treatment effects of RC versus BST for the primary outcome in pre-specified subgroups and for select secondary outcomes are shown, with missing data imputed as described in the methods.
Abbreviations: EORTC, European Organisation for Research and Treatment of Cancer Core Quality of Life (EORTC QLQ-C30).

eTable 10. Primary and key secondary patient-reported outcomes at baseline and 12 months by treatment group.

Outcome, mean (SD)	Baseline: Bladder-Sparing Therapy (BST)	Baseline: Radical Cystectomy (RC)	12 Months: Bladder-Sparing Therapy (BST)	12 Months: Radical Cystectomy (RC)	Group Difference (95% CI)
EORTC					
Physical Functioning[a]	88.6 (16.9)	89.8 (14.5)	85.0 (18.9)	86.5 (17.5)	-1.5 (-4.9, 1.9)
Male	88.4 (17.3)	90.9 (13.3)	84.4 (19.6)	87.1 (17.3)	-2.7 (-1.2, 6.5)
Female	89.7 (15.4)	85.6 (18.1)	87.0 (16.0)	84.1 (18.3)	2.9 (-10.2, 4.4)
Age ≥ 75	86.9 (17.1)	88.8 (14.8)	82.0 (20.2)	83.9 (17.4)	-1.9 (-4.0, 7.8)
Age < 75	89.8 (16.8)	90.3 (14.4)	87.0 (17.7)	87.7 (17.4)	-0.7 (-3.4, 4.8)
Has Spouse or Partner	90.0 (15.8)	91.1 (13.8)	86.0 (18.9)	87.3 (17.2)	-1.3 (-2.4, 4.9)
No Spouse or Partner	83.5 (19.9)	83.1 (16.8)	80.6 (18.7)	82.8 (17.4)	-2.3 (-6.7, 11.2)
CIS	89.9 (15.9)	90.1 (13.7)	83.3 (19.8)	86.5 (16.3)	-3.2 (-1.5, 8.0)
No CIS	87.5 (17.7)	89.2 (15.6)	86.4 (18.0)	86.6 (18.9)	-0.2 (-4.8, 5.2)
Global Health[a]	73.9 (18.2)	72.4 (19.6)	71.3 (19.9)	73.3 (18.5)	-2.0 (-5.6, 1.6)
Role Functioning[a]	87.2 (22.7)	86.3 (22.9)	82.7 (24.9)	83.8 (23.7)	-1.1 (-5.7, 3.5)
Emotional Functioning[a]	82.2 (18.3)	77.6 (20.3)	82.7 (18.2)	83.7 (18.9)	-0.9 (-4.5, 2.6)
Cognitive Functioning[a]	87.9 (17.2)	86.6 (18.4)	84.1 (19.0)	85.5 (16.2)	-1.4 (-4.7, 1.9)
Social Functioning[a]	84.8 (22.7)	81.4 (23.0)	83.2 (24.3)	80.6 (22.8)	2.6 (-1.8, 7.0)
Financial Difficulties[b]	12.4 (23.7)	13.9 (22.6)	14.6 (23.8)	10.3 (20.6)	4.4 (0.2, 8.5)
Fatigue[b]	20.1 (22.2)	21.9 (22.0)	25.8 (22.3)	26.8 (20.6)	-1.0 (-5.1, 3.0)
Nausea[b]	2.6 (8.9)	2.3 (7.1)	3.7 (10.5)	3.1 (8.9)	0.5 (-1.3, 2.3)
Pain[b]	16.7 (23.4)	17.1 (23.5)	17.0 (24.2)	16.1 (24.8)	0.9 (-3.7, 5.6)
Dyspnoea[b]	10.9 (20.7)	10.3 (19.3)	16.2 (24.1)	12.9 (19.6)	3.2 (-0.8, 7.3)
Insomnia[b]	22.5 (27.6)	29.5 (29.1)	26.3 (26.7)	26.3 (26.4)	0.0 (-5.0, 5.0)
Appetite[b]	7.8 (18.4)	8.6 (19.6)	11.2 (20.7)	9.9 (20.4)	1.2 (-2.6, 5.1)
Constipation[b]	10.1 (19.4)	10.2 (19.9)	15.1 (23.1)	18.4 (24.6)	-3.3 (-7.9, 1.2)
Diarrhoea[b]	8.3 (17.6)	8.8 (17.9)	8.4 (16.4)	11.9 (20.0)	-3.5 (-7.0, 0.1)
PROMIS Depression[b]	48.1 (8.2)	49.0 (8.0)	48.0 (8.3)	48.0 (8.2)	0.1 (-1.5, 1.6)
PROMIS Anxiety[b]	51.7 (9.3)	54.0 (8.8)	50.0 (8.9)	48.8 (8.9)	1.3 (-0.4, 3.0)
EQ-5D[a]	0.8 (0.1)	0.8 (0.1)	0.8 (0.1)	0.9 (0.1)	-0.0 (-0.0, 0.0)
COST[a]	30.4 (10.0)	30.3 (9.7)	30.4 (9.9)	31.0 (9.4)	-0.6 (-2.4, 1.2)
BCI[a]					
Urinary Summary	84.9 (15.5)	77.7 (19.7)	85.3 (16.7)	83.8 (14.2)	1.5 (-1.4, 4.4)
Urinary Function	85.8 (22.5)	77.7 (27.4)	80.2 (26.2)	80.5 (26.0)	-0.3 (-5.5, 4.9)
Urinary Bother	84.4 (16.5)	77.9 (19.7)	88.0 (15.7)	85.3 (14.1)	2.7 (-0.1, 5.6)
Bowel Summary	80.9 (9.2)	78.7 (9.4)	80.1 (9.5)	77.2 (10.8)	2.9 (0.9, 4.9)
Bowel Function	84.6 (16.7)	81.2 (17.9)	84.1 (15.4)	80.7 (18.5)	3.4 (0.0, 6.8)
Bowel Bother	78.6 (8.3)	77.5 (8.9)	77.4 (9.7)	75.2 (10.6)	2.2 (0.2, 4.2)
Sexual Summary	49.3 (25.5)	48.2 (26.6)	47.1 (25.7)	32.6 (20.1)	14.5 (9.9, 19.2)
Sexual Function	36.8 (27.8)	38.0 (29.2)	34.9 (28.7)	21.2 (22.4)	13.6 (8.5, 18.8)
Sexual Bother	66.2 (30.4)	62.2 (29.3)	64.6 (29.3)	48.8 (25.6)	15.8 (10.2, 21.4)

Abbreviations: EORTC, European Organisation for Research and Treatment of Cancer Core QLQ-C30; CIS, *carcinoma in situ*; PROMIS, Patient Reported Outcome Measurement Information System; EQ-5D: EuroQoL EQ-5D-5L; COST: Comprehensive Score for Financial Toxicity; BCI, Bladder Cancer Index.

[a]Higher score means better functioning / health / quality of life

[b]Higher score means worse symptoms

eTable 11. Average treatment effects for each outcome using GEE as a sensitivity analysis.

Outcome	Treatment Effect (95% CI)
EORTC	
Physical Functioning[a]	0.90 (-1.81, 3.61)
Male	-0.57 (-16.61, 15.48)
Female	1.28 (-1.70, 4.26)
Age ≥ 75	0.11 (-9.21, 9.43)
Age < 75	1.10 (-2.34, 4.54)
Has Spouse or Partner	0.73 (-3.48, 4.94)
No Spouse or Partner	1.82 (-5.20, 8.83)
CIS	2.49 (-3.71, 8.69)
No CIS	-0.49 (-4.65, 3.66)
Global Health[a]	1.48 (-1.57, 4.53)
Role Functioning[a]	2.38 (-2.13, 6.88)
Emotional Functioning[a]	4.03 (1.09, 6.97)
Cognitive Functioning[a]	1.79 (-1.07, 4.65)
Social Functioning[a]	-0.63 (-5.37, 4.12)
Financial Difficulties[b]	-6.00 (-10.56, -1.44)
Fatigue[b]	-0.44 (-4.09, 3.21)
Nausea[b]	-1.39 (-3.37, 0.59)
Pain[b]	-1.76 (-5.80, 2.29)
Dyspnoea[b]	-3.06 (-7.32, 1.20)
Insomnia[b]	-2.85 (-7.43, 1.73)
Appetite[b]	-3.69 (-7.80, 0.42)
Constipation[b]	2.54 (-2.22, 7.29)
Diarrhoea[b]	2.94 (-0.73, 6.61)
PROMIS Depression[b]	-1.18 (-2.49, 0.12)
PROMIS Anxiety[b]	-2.39 (-3.83, -0.96)
EQ-5D[a]	0.03 (0.01, 0.05)
COST[a]	1.46 (-0.09, 3.02)
BCI[a]	
Urinary Summary	1.33 (-1.55, 4.22)
Urinary Function	4.30 (-0.70, 9.29)
Urinary Bother	-0.02 (-2.95, 2.92)
Bowel Summary	-1.46 (-3.30, 0.37)
Bowel Function	-1.81 (-4.89, 1.27)
Bowel Bother	-0.95 (-2.87, 0.98)
Sexual Summary	-11.51 (-15.67, -7.36)
Sexual Function	-12.80 (-17.94, -7.65)
Sexual Bother	-9.67 (-15.04, -4.30)

Abbreviations: EORTC, European Organisation for Research and Treatment of Cancer Core QLQ-C30; CIS, *carcinoma in situ*; PROMIS, Patient Reported Outcome Measurement Information System; EQ-5D: EuroQoL EQ-5D-5L; COST: Comprehensive Score for Financial Toxicity; BCI, Bladder Cancer Index.

[a]Higher score means better functioning / health / quality of life

[b]Higher score means worse symptoms

eTable 12. Treatment arm-specific clinical outcomes 12 months after recurrence of high-grade non-muscle invasive bladder cancer

Event, n (%)	Bladder-Sparing Therapy (BST)	Radical Cystectomy (RC)
**Arm-Specific Clinical Outcomes**		
Eventual cystectomy	27/334 (8.1%)	n/a
Due to recurrence	21/334 (6.3%)	n/a
Due to progression	4/334 (1.2%)	n/a
Due to other cause	2/334 (0.6%)	n/a
Upstaging at cystectomy	n/a	44/198 (22%)
Prolonged hospitalization following cystectomy	n/a	15/187 (8.0%)
Any hospitalization within 90 days of cystectomy	n/a	57/193 (30%)
Death within 90 days of cystectomy	n/a	5/198 (2.5%)

eTable 13. Counts of adverse events categorized by grade reported within 12 months of study enrollment by arm.

Event[a]	Arm	1	2	3	4	5	Unknown	Total
General	BST	24	10	2	0	0	4	40
	RC	21	11	4	0	0	2	38
Gastrointestinal	BST	21	14	10	0	0	0	45
	RC	34	40	36	2	0	0	112
Infectious	BST	0	1	8	4	0	0	13
	RC	0	1	33	4	2	0	40
Wound	BST	1	0	0	0	0	0	1
	RC	6	5	8	2	0	0	21
Genitourinary	BST	35	66	31	0	0	0	132
	RC	8	41	53	1	0	3	106
Cardiac	BST	3	2	7	0	1	0	13
	RC	4	6	3	0	0	0	13
Pulmonary	BST	3	11	5	2	1	0	22
	RC	1	7	3	1	1	0	13
Bleeding	BST	4	5	4	0	1	0	14
	RC	14	16	20	0	0	0	50
Thromboembolic	BST	0	4	2	0	0	0	6
	RC	0	3	9	0	0	0	12
Neurological	BST	1	5	3	0	0	0	9
	RC	5	5	0	0	0	0	10
Endocrine	BST	1	1	0	0	0	0	2
	RC	0	0	0	0	0	0	0
Other	BST	0	0	0	0	11	7	18
	RC	0	0	0	0	8	25	33
Unknown	BST	0	0	0	0	0	1	1
	RC	0	0	0	0	0	3	3
Total	BST	93	119	72	6	14	12	316
	RC	93	135	169	10	11	33	451

[a]AE grades for each type are defined by the Common Terminology Criteria for Adverse Events (CTCAE) Version 5.0.[9]

eTable 14. Proportion of affected participants by arm that experienced an adverse event (AE) or serious adverse event (SAE) by organ system.

Event Type, n (%)	Grade[a]	Bladder-Sparing Therapy (BST)	Radical Cystectomy (RC)	Weighted RR for RC vs. BST (95% CI)[b]
General	1-3 (AE)	35 (9%)	27 (14%)	1.37, (0.84 - 2.23)
	4-5 (SAE)	0 (0%)	0 (0%)	n/a
Gastrointestinal	1-3 (AE)	31 (8%)	62 (31%)	3.75, (2.49 - 5.63)
	4-5 (SAE)	0 (0%)	2 (1%)	n/a
Infectious	1-3 (AE)	8 (2%)	25 (13%)	5.48, (2.46 - 12.20)
	4-5 (SAE)	4 (1%)	5 (3%)	1.84, (0.49 - 6.89)
Wound	1-3 (AE)	1 (0%)	16 (8%)	24.10, (3.19 - 182.06)
	4-5 (SAE)	0 (0%)	2 (1%)	n/a
Genitourinary	1-3 (AE)	87 (23%)	58 (29%)	1.20, (0.89 - 1.60)
	4-5 (SAE)	0 (0%)	1 (1%)	n/a
Cardiac	1-3 (AE)	11 (3%)	12 (6%)	2.01, (0.88 - 4.57)
	4-5 (SAE)	1 (0%)	0 (0%)	n/a
Pulmonary	1-3 (AE)	14 (4%)	10 (5%)	1.49, (0.67 - 3.34)
	4-5 (SAE)	3 (1%)	2 (1%)	0.91, (0.15 - 5.51)
Bleeding	1-3 (AE)	12 (3%)	42 (21%)	6.30, (3.35 - 11.85)
	4-5 (SAE)	1 (0%)	0 (0%)	n/a
Thromboembolic	1-3 (AE)	6 (2%)	12 (6%)	3.23, (1.21 - 8.63)
	4-5 (SAE)	0 (0%)	0 (0%)	n/a
Neurological	1-3 (AE)	9 (2%)	10 (5%)	1.95, (0.79 - 4.81)
	4-5 (SAE)	0 (0%)	0 (0%)	n/a
Endocrine	1-3 (AE)	2 (1%)	0 (0%)	n/a
	4-5 (SAE)	0 (0%)	0 (0%)	n/a
Other	1-3 (AE)	7 (2%)	18 (9%)	4.84, (2.02 - 11.56)
	4-5 (SAE)	11 (3%)	8 (4%)	1.24, (0.50 - 3.09)
Unknown	1-3 (AE)	1 (0%)	3 (2%)	4.48, (0.46 - 43.79)
	4-5 (SAE)	0 (0%)	0 (0%)	n/a
Total	1-3 (AE)	140 (38%)	123 (62%)	1.61, (1.35 - 1.91)
	4-5 (SAE)	18 (5%)	16 (8%)	1.68, (0.84 - 3.39)

[a]Events of unknown grade were included in grades 1-3
[b]Inverse probability weighted risk ratios (RR) calculated using quasi-Poisson regression

References

1. Chang S, Boorjian S, Chou R, et al. Diagnosis and Treatment of Non-Muscle Invasive Bladder Cancer: AUA/SUO Joint Guideline (2020). Accessed September 25, 2021. https://www.auanet.org/guidelines/guidelines/bladder-cancer-non-muscle-invasive-guideline

2. Kind AJH, Buckingham WR. Making Neighborhood-Disadvantage Metrics Accessible — The Neighborhood Atlas. *N Engl J Med*. 2018;378(26):2456-2458. doi:10.1056/NEJMp1802313

3. USDA ERS - Documentation. Accessed May 28, 2024. ers.usda.gov/data-products/rural-urban-commuting-area-codes/documentation/

4. Zang Y, Li X, Cheng Y, Qi F, Yang N. An overview of patients with urothelial bladder cancer over the past two decades: a Surveillance, Epidemiology, and End Results (SEER) study. *Ann Transl Med*. 2020;8(23):1587. doi:10.21037/atm-20-2108

5. Iyer I, Zhang S, Borno H. Evaluating therapeutic bladder cancer trial disparities in race/ethnicity. *J Clin Oncol*. 2022;40(6_suppl):446-446. doi:10.1200/JCO.2022.40.6_suppl.446

6. Fang W, Yang ZY, Chen TY, Shen XF, Zhang C. Ethnicity and survival in bladder cancer: a population-based study based on the SEER database. *J Transl Med*. 2020;18(1):145. doi:10.1186/s12967-020-02308-w

7. Bladder cancer statistics. World Cancer Research Fund. Accessed April 4, 2025. https://www.wcrf.org/preventing-cancer/cancer-statistics/bladder-cancer-statistics/

8. Rural-Urban Commuting Area Codes | Economic Research Service. Accessed June 5, 2025. https://www.ers.usda.gov/data-products/rural-urban-commuting-area-codes

9. Common Terminology Criteria for Adverse Events (CTCAE) Version 5.0. U.S. Department of Health and Human Services. Accessed June 5, 2025. https://ctep.cancer.gov/protocoldevelopment/electronic_applications/docs/ctcae_v5_quick_reference_5x7.pdf
