# Supplementary material for: Twelve-Month Results From the CISTO Study Comparing Radical Cystectomy Versus Bladder-Sparing Therapy for Recurrent High-Grade Non–Muscle-Invasive Bladder Cancer
Source: J Clin Oncol. 2025 Dec 15;44(4):274–85. doi: 10.1200/JCO-25-01324 (PMC12707586; doi:10.1200/JCO-25-01324)
Supplement: Supplementary file 3 [file jco-44-274-s003.pdf]

The Comparison of Intravesical Therapy and Surgery as Treatment Options (CISTO) for Bladder Cancer Study

Co-Directors John L. Gore, MD MS and Angela B. Smith, MD MS

Study Funder:

Patient Centered Outcomes Research Institute (Principal Investigator-Gore)

Version Number and Date:

V7.0 – April 4, 2023

Table of Contents

1.0 Key Personnel 4
2.0 Background and Significance 5
2.1 Rationale 5
3.0 Study Aims 5
4.0 Administrative Organization 6
4.1 Research Activity Coordination 6
4.2 Participating Sites 6
5.0 Study Design 6
5.1 Study Overview 6
5.2 Study Population 7
5.3 Participant Screening and Enrollment 7
5.4 Patient Participant Selection 7
5.5 Caregiver Eligibility 8
5.6 Consent Process 8
6.0 Study Assessments and Activities 9
6.1 Study Schedule Overview 9
6.2 Patient Baseline Assessment 10
6.3 Patient Follow-up Assessments 11
6.4 Subset of Patients and Caregivers 11
6.5 Participant Burnout and Optimizing Data Collection 11
6.6 Electronic Medical Record Abstraction 11
6.7 Participant Withdrawal 12
6.8 Compensation 12
7.0 Outcome Assessments 12
7.1 Primary Outcome 12
7.2 Secondary Outcomes 13
8.0 Data Management and Information Security 13
8.1 Data Management 13

8.2 Information Security 13

8.3 Subset of Patients and Caregivers 14

9.0 Statistical Analysis Plan 14

9.1 Delineation of Treatment Arms 14

9.2 Analytic Plan 14

9.3 Missing Data 16

9.4 Sample Size and Statistical Power 16

10.0 Data and Safety Monitoring Plan 17

10.1 SAE Reporting 18

11.0 Data Quality and Performance Management 20

11.1 Site Training 20

11.2 Quality Management Plan 20

11.3 Performance Management Plan 21

11.4 Protocol Violations 21

12.0 Protection of Human Participants 22

13.0 Ancillary Studies 22

14.0 References 23

1.0 Key Personnel

Principal Investigators	John L. Gore, MD MS<br>University of Washington<br>Department of Urology<br>1959 NE Pacific Street, Box 356410<br>Seattle, WA 98195<br>Phone: 206-221-6430<br>Email: jlgore@uw.edu
	Angela B. Smith, MD MS<br>University of North Carolina<br>Department of Urology<br>2115 Physicians Office Building<br>Chapel Hill, NC 27599-7235<br>Phone: 919-966-8217<br>Email: angela_smith@med.unc.edu
Director, Data Coordinating Center	Bryan Comstock, MS<br>University of Washington<br>Department of Biostatistics<br>Phone: 206-543-1882<br>Email: bac4@uw.edu
Director, Clinical Coordinating Center	Erika Wolff, PhD<br>University of Washington<br>Phone: 206-221-3174<br>Email: ewolff2@uw.edu
Director, Stakeholder Coordinating Center	Angela B. Smith, MD MS<br>University of North Carolina<br>Department of Urology<br>2115 Physicians Office Building<br>Chapel Hill, NC 27599-7235<br>Phone: 919-966-8217<br>Email: angela_smith@med.unc.edu
Project Manager	Kristin Follmer<br>University of Washington<br>Phone: 206-685-8708<br>Email: kfollmer@uw.edu
Biostatistician	Michael Nash, MS<br>University of Washington<br>Department of Biostatistics<br>Phone: 206-543-2589<br>Email: mgnash@uw.edu

2.0 Background and Significance

Bladder cancer is the most common urinary tract cancer, affecting men and women, and is the 5th most common cancer in the US.[1] Yet bladder cancer research is underfunded relative to other common cancers.[2] As a result, bladder cancer care is prone to evidence gaps that produce decision uncertainty for both patients and clinicians. The Comparison of Intravesical Therapy and Surgery as Treatment Options (CISTO) for Bladder Cancer Study has the potential to fill these critical evidence gaps, change care pathways for the management of NMIBC (non-muscle-invasive bladder cancer), and provide for personalized, patient-centered care. The purpose of CISTO is to conduct a large prospective study that directly compares the impact of medical management versus radical cystectomy in recurrent high-grade NMIBC patients with BCG (Bacillus Calmette-Guerin) failure on clinical outcomes and patient and caregiver experience using standardized patient-reported outcomes (PROs).

2.1 Rationale

Most bladder cancer patients (74%) present with NMIBC where the cancer is limited to the lining or support layer of the bladder.[3] High-grade NMIBC is treated initially with endoscopic resection and intravesical immunotherapy, followed by bladder instillations of BCG.[3–5] Most patients with high-risk, high-grade NMIBC are able to retain their bladders and avoid more invasive treatments. However, 24-61% of patients will have their cancers recur within 12 months of treatment with BCG (BCG failures), and they have limited treatment options.[6] National guidelines recommend consideration between two alternatives: additional medical management and radical cystectomy (removal of the bladder).[5] Selecting between these options involves weighing the risk of progression of bladder cancer and loss of a window of potential cure versus the risk of morbidity and loss of quality of life (QOL) with bladder removal. This complex decision-making engages patients and their caregivers, who may be impacted by the urinary, sexual, and bowel dysfunctions that can occur with NMIBC treatment. We anticipate identifying which patients for whom radical cystectomy represents the best option, and similarly which patients are best treated with medical management.

We will assess the comparative effectiveness of medical management versus radical cystectomy in recurrent high-grade NMIBC patients where first-line treatment, usually BCG, failed. We will evaluate our research question on a large scale in real world practice settings including academic and community-based practices and examine patient-centered outcomes. We include a community urologist on our Executive Committee to ensure that the results are relevant to, and will be disseminated back to, community urology practice. Additionally, we have engaged stakeholders with diverse perspectives relevant to this research question, including patients, caregivers, national patient advocacy organizations, national medical specialty organizations, guideline developers, health care payers, and industry. By engaging broad expertise relevant to this research question, we will ensure that the study results will help NMIBC patients whose cancer recurs after treatment make more informed decisions that improve the health outcomes that are important to them. By engaging national bladder cancer experts, community urologists, and guideline developers, we have high potential for our study results to be adopted into clinical practice.

3.0 Study Aims

CISTO is a multi-site prospective observational cohort study of patients whose high-grade NMIBC has recurred after first-line intravesical BCG.

Aim 1: To compare patient-reported and patient-centered clinical outcomes between patients

undergoing radical cystectomy and those receiving medical management for NMIBC that have failed first-line BCG.
Hypothesis 1.1: Patients undergoing radical cystectomy will have worse generic health-related QOL within 12 months of bladder removal surgery compared with patients who choose medical management.
Hypothesis 1.2: 12-month disease-free survival and metastasis-free survival will be better among radical cystectomy patients than among patients who choose medical management.
Hypothesis 1.3: Patient-reported and clinical outcome differences will vary within important subgroups including women, non-white patients, elderly patients, patients with multiple comorbid health conditions, patients with poor urinary function scores at the time of BCG failure, patients without caregivers, patients with atypical cancer histologies, and patients receiving investigational agents.

Aim 2: To characterize the heterogeneity of treatments received and corresponding patient and caregiver preferences for NMIBC that is not responsive to first-line treatment.
Hypothesis 2.1: Patients who choose radical cystectomy for NMIBC after first line treatment will do so more often because they feel this offers their best chance for survival. Patients who choose medical management will do so more often because they are concerned about the complications and QOL detriments associated with radical cystectomy.
Hypothesis 2.2: Patient and caregiver preferences will correlate strongly with observed treatments received and health-related QOL outcomes. We anticipate that QOL will vary by concordance of treatments received with treatment preferences and health state utilities.

4.0 Administrative Organization

The University of Washington hosts several projects and programs to monitor risks, benefits, and value of health care treatments and to determine whether these developments improve quality of care and the health outcomes of patients.[7]

4.1 Research Activity Coordination

The Clinical Coordinating Center (CCC) and Stakeholder Coordinating Center (SCC) are located within the University of Washington School of Medicine. The UW Center for Biomedical Statistics will serve as the Data Coordinating Center (DCC).

4.2 Participating Sites

The CCC will coordinate the trial. Investigators will coordinate the study at the University of Washington Medical Center (UWMC) and other clinical sites. In preparing for the study, 32 potential hospital sites were engaged and considered for involvement in the study based on readiness of site infrastructure, staff engagement, patient population characteristics, and number of recurrent high-grade NMIBC cases. Additional sites may be recruited for participation given the targeted volume and rate of participant accrual.

5.0 Study Design

5.1 Study Overview

CISTO is a prospective, non-randomized, observational cohort study of people with NMIBC that has recurred after intravesical BCG treatment. We will compare patients who choose medical management such as BCG, chemotherapy, or investigational therapies, with patients undergoing radical cystectomy.

5.2 Study Population

The CISTO study will include 900 patient participants and 25 caregivers as participants. Patients with a diagnosis of recurrent high-grade NMIBC which did not respond to first-line BCG and who are considering second-line treatment will be approached for participation in this observational study. Enrollment at each site will aim for at most a 2:1 ratio of participants selecting medical management to radical cystectomy in order to ensure adequate enrollment of radical cystectomy patients. There will also be a qualitative sub-study that will include interviews of 50 patients and 25 caregivers recruited from the observational cohort study.

5.3 Participant Screening and Enrollment

Patients will be screened by study investigators and/or study coordinators based on alerts from clinicians, staff, or screening of urology and/or cancer care practice logs. Patients will be identified as potential candidates for the study based on inclusion and exclusion criteria collected as part of standard care, including a confirmatory pathology report. Patients will be asked to provide contact information for their caregiver. Caregivers will only be approached for consent for patients who have consented to participating in the CISTO study.

The screening form for the CISTO study will be completed for patients with a diagnosis of recurrent high-grade NMIBC that have failed intravesical therapy. Completion and data-entry of this form is for all patients who are eligible for and have been approached for study participation. Reasons for refusal of study participation will be collected.

Sites are responsible for maintaining their own screening log in accordance with their institutional policies and guidelines. This log should document adequate information such that the research team may disclose to the institutional compliance department that a patient’s electronic medical record (EMR) was accessed to screen for potential eligibility for the CISTO study. UW Medicine requires disclosure of accessing the EMR for purposes other than treatment, payment, and health care operations.

Sites are required to periodically provide a de-identified census of recurrent high-grade NMIBC patients to the UW CCC. This census will be compared with the number of participants reported as screened at each site and will serve as a measure of protocol compliance.

5.4 Patient Participant Selection

5.4.1 Inclusion Criteria

1. Adult 18 years of age or older; and
2. Presenting with high-grade NMIBC established by anatomic pathology as tumor stage classification Tis, Ta, or T1, and with:
    a. Pathology documentation from any hospital/clinic/medical center, and
    b. More than 50% urothelial carcinoma component in the specimen
3. History of high-grade NMIBC established by anatomic pathology as tumor stage classification Tis, Ta, or T1; and
4. Attempted or received induction BCG (at least 3 out of 6 instillations) at any point in time; and
5. In the previous 12 months, received at least one instillation of any intravesical agent (induction or maintenance) or one administration of systemic therapy for NMIBC treatment.

5.4.2 Exclusion Criteria

Participants must not have any of the following exclusion criteria:

1. Any plasmacytoid or small cell (neuroendocrine) component in the pathology (past or current presentation);
2. Previous history of cystectomy or radiation therapy for bladder cancer;
3. Previous history of muscle-invasive bladder cancer or metastatic bladder cancer;
4. Any history of upper tract urothelial carcinoma;
5. Incarcerated in a detention facility or in police custody (patients wearing a monitoring device can be enrolled) at baseline/screening;
6. Contraindication to radical cystectomy (e.g., ASA of 4, patient not considered a radical cystectomy candidate due to comorbidity);
7. Contraindication to medical management (i.e., intolerant of all medical therapies);
8. Unable to provide written informed consent in English;
9. Unable to be contacted for research surveys;
10. Planning to participate in a Phase I or Phase II interventional clinical trial for NMIBC (unless in the control/comparator arm of a Phase II trial) or any blinded interventional trial for NMIBC.

5.5 Caregiver Eligibility

5.5.1 Inclusion Criteria

1. Adult 18 years of age or older

5.5.2 Exclusion Criteria

Participants must not have any of the following exclusion criteria:

1. Unable to provide written informed consent in English;

5.6 Consent Process

The research coordinator and a representative from the medical team will confirm the patient's eligibility for the study based on inclusion and exclusion criteria. The research coordinator or medical team representative may approach the patient in the clinic or use a remote method such as telephone or videoconferencing.. The patient will be provided the opportunity to review informational materials related to the study, which may include an informational video. The Research Coordinator will review the informed consent document with the patient; this will include reviewing the purpose of the study, study procedures, the risks and benefits of study participation and alternatives to study participation. Adequate time will be allowed for patients to ask questions and have these questions answered to their full satisfaction. Finally, patients will be asked to participate.

Patients willing to participate may need more time to decide on their clinical treatment plan with their provider. In these cases, the research coordinator may ask the patient for permission to re-approach for the

study after the clinical decision has been made. Alternatively, patients may be willing to participate but their treatment arm has capped enrollment. Patients may agree to be contacted by the research coordinator if enrollment slots become available.

All patients agreeing to participate will be asked to sign the informed consent form (ICF) either electronically or on paper and will be given a copy for their records. The HIPAA Authorization form will also be signed and a copy given to the patient for their records, following IRB guidance. For some partnering sites, HIPAA language will be incorporated into the informed consent document but for others, this will be a separate document.

5.6.1 Subset of Patients and Caregivers

A subset of 50 patients and 25 caregivers will also be asked to participate in a qualitative interview. Written consent for all study activities for the patient will be obtained at the time of initial enrollment in the study. At the time of recruitment for interviews, participants will be informed that, as with all study activities, their participation in this portion of the study is voluntary and they may opt out of the interview at any time. The addition of interviews will not have any impact on the risks or benefits for study participants.

At study enrollment, the patient will be asked to provide information about the presence of a caregiver, the relationship between the patient and the caregiver, and caregiver contact information. Caregivers will be approached for participation in the caregiver interview if the patient has consented to participate in this study and gives permission for the study team to contact the caregiver.

The research coordinator will confirm the caregiver's eligibility for the study based on inclusion and exclusion criteria. Upon approaching the caregiver (in person, telephone, email, or mail), the caregiver will be provided the opportunity to review informational materials related to the study, which may include an informational video. The Research Coordinator will review the informed consent document with the caregiver; this will include reviewing the purpose of the study, study procedures, the risks and benefits of study participation and alternatives to study participation. Adequate time will be allowed for caregivers to ask questions and have these questions answered to their full satisfaction. Finally, caregivers will be asked to participate. All caregivers agreeing to participate will be asked to sign the ICF either electronically or on paper and will be given a copy for their records.

6.0 Study Assessments and Activities

6.1 Study Schedule Overview

Consenting participants will be asked to complete research assessments on the topics and at the time points described in ***Table 1*** and ***Table 2***.

Table 1. Overview of Patient Study Assessments

Patient Assessments	Time Point										
	Year 1					Year 2		Year 3		Year 4	
	BL	M3	M6	M9	M12	M18	M24	M30	M36	M42	M48
Demographics	X										
Comorbid health conditions	X				X		X		X		X
Single Item Literacy Screener[8]	X										
Exposure questions[9]	X										
Bladder cancer history	X										
EORTC QLQ C30	X	X	X	X	X	X	X	X	X	X	X
Bladder cancer index	X	X	X	X	X	X	X	X	X	X	X
COST financial toxicity measure	X				X		X		X		X
PROMIS anxiety scale	X	X	X	X	X	X	X	X	X	X	X
PROMIS depression scale	X	X	X	X	X	X	X	X	X	X	X
General health history (healthcare utilization)		X	X	X	X	X	X	X	X	X	X
Decision regret					X		X		X		X
Generic QOL (EQ-5D-5L) and preferences for health states relevant to NMIBC that have failed first-line BCG (TTO)	X				X						

Table 2. Overview of Patient Assessments for Extended Follow-up

Patient Assessments	Time Point					
	Year 5	Year 6	Year 7	Year 8	Year 9	Year 10
	M60	M72	M84	M96	M108	M120
General health history	X	X	X	X	X	X
EORTC QLQ C30	X	X	X	X	X	X
Bladder cancer index	X	X	X	X	X	X
PROMIS anxiety scale	X	X	X	X	X	X
PROMIS depression scale	X	X	X	X	X	X

To ensure successful retention of participants and completion of research assessments through the four-year and extended follow-up periods, detailed contact information including name, address, phone numbers, email addresses, as well as alternate contact persons and phone numbers will be requested from participants at baseline (and updated at subsequent time points). Although complete contact information is ideal to optimize future retention, only one means of contacting the participant is required for enrollment.

6.2 Patient Baseline Assessment

The patient baseline assessment should be completed in-person with the site research coordinator, or may be completed online, by phone, or by mail, as needed. The measures are listed in Table 1.

6.3 Patient Follow-up Assessments

The patient follow-up assessments should be completed in-person, online, by phone, or by mail. The measures included at each time point are listed in Tables 1 and 2, if participating in the extended/long-term follow-up. UW study staff will contact the participant to complete the follow-up assessments. Outreach will include a combination of phone, mail, and email as determined by the contact information provided by the participant. If the contact protocol is exhausted and the assessment has not been completed, a research coordinator at the participant's enrollment site will be notified. They will review the EMR and provide any site-specific or EMR-specific updates that may be helpful; for example, an updated telephone number. Additional contact attempts (calls, emails, texts, etc.) may be attempted from the local site coordinators. If the assessment is still not completed, it will be considered missed and attempts to contact the participant will resume at the next assessment time point.

6.4 Subset of Patients and Caregivers

A subset of 50 patients and 25 caregivers will be asked to participate in qualitative interviews. Participants will be recruited across each of the study sites and will include patients and caregivers of patients undergoing radical cystectomy and medical management. Interview participants will be approached at least 6 months but no more than 24 months after enrollment in CISTO.

Due to the geographical spread of study site locations, interviews will be conducted using a HIPAA-compliant teleconference platform. Study team members with training and expertise in qualitative research will conduct the interviews. The interviewer will review the information sheet prior to beginning the interview, verbally confirm that the participant agrees to participate, understands the nature of the interview, and understands they can choose not to participate in an interview without affecting continued participation in the overall study. Interviews will utilize one of two semi-structured interview guides, one for patient interviews and another for caregiver interviews. Interviews will last approximately 60 minutes and will be audio recorded and transcribed.

6.5 Participant Burnout and Optimizing Data Collection

Participants may experience research assessment burnout due to the frequency and number of questions asked of them. To optimize complete data collection, research personnel should recognize a participant's reluctance to complete the research assessment and request that the participant instead complete a subset of survey responses (minimal research assessment). In the event that a participant requests to complete a minimal research assessment, research personnel should prioritize asking the research participant questions in the priority order listed in Tables 1 and 2. The research coordinator should use their best judgment to determine how many additional survey questions should be asked. It is preferred to miss some responses at one research assessment time point rather than risk the participant withdrawing altogether due to research assessment burnout.

6.6 Electronic Medical Record Abstraction

Patients consenting to the study will have their EMR reviewed at pre-determined time points. Research coordinators will conduct an EMR review at baseline and for subsequent healthcare utilization events and clinical outcomes. Data collected will include variables relating to health history, transurethral resection of a bladder tumor (TURBT), medical management, laboratory values, imaging results, pathology findings, cystectomy, and adverse events.

6.7 Participant Withdrawal

6.7.1 Reasons for Withdrawal

Participants may be withdrawn from the study if the participant desires withdrawal and will notify the local site research team or the UW research team; reasons for withdrawal will be recorded.

6.7.2 Handling of Withdrawals

If the participant requests to be withdrawn from the study, they will not continue with scheduled study follow-ups. If the participant withdraws from the study and withdraws consent for disclosure of future information, no further evaluations will be performed, and additional data will not be collected. The investigators may retain and continue to use data collected before withdrawal of consent.

If the patient participant withdraws from the study, the caregiver participant will not be contacted.

6.8 Compensation

6.8.1 Participant Compensation

Participants will be paid for completing the research assessments described in Tables 1 and 2. Patients will be compensated $25 for the baseline assessment, and $25 for each of the follow-up assessments at 3, 6, 9, 12, 18, 24, 30, 36, 42, and 48 months. Compensation will be distributed to participants centrally by UW research staff unless other arrangements have been made.

For participants in the extended follow-up cohort, payment will continue with $25 for each survey completed.

6.8.2 Compensation for Subset of Patients and Caregivers

The subset of patients and caregivers participating in the qualitative interviews will be compensated $50 for completing the interview.

6.8.3 Site and Site Lead Compensation

Each site will determine how best to allocate their funds to support this research at their hospital. Funds may be used to support study staff including site surgical and lead investigators, relevant clinical and research support staff, and research study supplies. Funds for research participant compensation will not come directly from these site payments but rather provided by UW centrally as described in Section 6.8.

7.0 Outcome Assessments

7.1 Primary Outcome

The *primary outcome* is patient-reported health-related quality of life as measured with the EORTC QLQ-C30,[10] which has been previously used in bladder cancer populations. The *primary endpoint* is the physical functioning domain score of the EORTC QLQ-C30 at 12-months.

7.2 Secondary Outcomes

For patients, secondary outcomes include self-reported urinary and sexual function measured with the Bladder Cancer Index,[11] NMIBC treatment preferences, decisional regret, financial distress measured with the Comprehensive Score for Financial Toxicity (COST),[12] healthcare utilization measured as 12-month hospital and urology clinic days, return to work/normal activities, 12-month disease-free survival, metastasis-free survival, bladder cancer-specific survival, and progression to muscle-invasive bladder cancer. Among patients with available data, we will assess health-related quality of life, health literacy, urinary function, sexual function, financial distress, healthcare utilization, disease-free and metastasis-free survival, bladder cancer-specific survival, and progression at up to 48 months, and up to 6 additional years for extended follow-up.

8.0 Data Management and Information Security

8.1 Data Management

The UW DCC will host a public facing web page that provides a centralized location for information about the research project for patients, investigators, and institutional agencies. The public-facing web page contains a link to an https-secured web-based portal (https://uwdcc.org/portal/cisto) that provides a centralized location to securely perform study data management activities using individual Single Sign on (SSO) user names and passwords sponsored by the UW DCC.

The UW DCC supports an installation of REDCap (Research Electronic Data Capture: http://www.projectredcap.org), which is software specifically designed for electronic data capture in research studies. All data entered are site-specific and not available to personnel at other recruitment sites. Investigators with UW DCC research studies have the flexibility to collect data through mailed paper-based surveys, computer assisted phone-based interviews, SMS messages, or with web-based surveys. For most studies, we also provide customized statistical reporting and C#, .NET, and R programming beyond what REDCap provides natively. Data systems are interlinked through the REDCap application programming interface (API).

8.2 Information Security

All participant data collected on paper at sites will be stored in locked file cabinets located in the site research offices.

Electronic data captured under the CISTO protocol will be stored and accessible only through REDCap and will not be stored locally on laptops or tablets. Additionally, protected health identifiers will be stored in a REDCap database without data export rights enabled. Data stored on the University of Washington REDCap servers are only accessible to allowed study personnel through SSO authentication procedures. Many institutions that have installed REDCap have made use of enterprise-level web application security scanners, such as HP Webinspect and Acunetix, to scan and test REDCap's security and its ability to withstand various methods of attack. REDCap has performed very well in such instances.

Access to research data collected in each UW DCC study is restricted to study team members at each site and appropriate UW CCC and DCC personnel. Protected Health Information (PHI) from clinical recruitment sites are protected by virtual machine servers and stored in server room that meets the technical requirements for Health Insurance Portability and Accountability Act (HIPAA) compliance.

8.3 Subset of Patients and Caregivers

For the qualitative interviews obtained from a subset of patients and caregivers, only the research team will access the interview data, including transcripts and any field notes produced during interviews. Data will be kept in a password-protected database. Audio recordings may include identifying information (such as name or title), but these will be removed from the transcripts of those files and the original audio files will be destroyed. We will keep audio recordings until a transcript has been created and reviewed for accuracy. As is consistent with other approved research activities, all other records (e.g., transcripts, field notes) will be de-identified, and will not contain participant name or any other identifying information.

Presentations of findings, such as conference papers, will present most data in aggregate form. Qualitative feedback may be presented as participant quotes, and care will be taken by the research team to select generalized quotes that minimize disclosure of an individual participant by excluding names, locations, organizations, and other cues to participant identity.

9.0 Statistical Analysis Plan

9.1 Delineation of Treatment Arms

We will use block recruitment within practice sites, no more than two thirds of whom within each completed block may be medical management patients. Using block recruitment ensures that the study will be sufficiently well balanced to adequately characterize the effect of each treatment relative to the other and maintain power to evaluate primary and secondary outcomes. Each treatment arm is defined by each patient's choice of treatment at enrollment. If a patient ultimately receives a different treatment from what they chose initially, this is considered as an outcome of their initially chosen treatment. We expect that all or almost all patients who choose radical cystectomy will receive it prior to evaluation of the primary endpoint at 12 months post-enrollment, while up to 20% of patients who choose medical management may undergo radical cystectomy during this period.

9.2 Analytic Plan

We will use descriptive statistics to characterize the treatments received, clinical outcomes and PROs, and patient and caregiver participant preferences. We will compare continuous demographic and clinical characteristics by treatment group with nonparametric tests to protect against violations of normality assumptions. Exact p-values from Exact Conditional Tests, such as Fisher's exact test and its multi-degree of freedom extensions, will be used to compare categorical data.

Aim 1 hypotheses relate to the effect of the two treatments (radical cystectomy and medical management) on three clinical outcomes (health-related QOL, disease-free survival, and metastasis-free survival). Treatment selection bias is an inherent part of any pragmatic observational study design. To address potential confounding by indication in the comparison of radical cystectomy and those receiving intravesical therapies, we will utilize a novel state-of-the-art method called targeted maximum likelihood estimation (TMLE) as our primary analytic approach.[13–15] TMLE is an innovative analytic method for causal effect estimation using observational data. Unlike general propensity score-based or G-computation approaches, TMLE is doubly robust to misspecification arising from an omitted confounding variable in the models for the exposure (treatment) or the outcome measure. Unlike approaches that focus on creating carefully matched treatment and control groups, *TMLE allows for the inclusion of all patient participants and their reported outcome measures*.[13–15]

TMLE uses a prediction model such as covariate-adjusted regression to generate an initial estimate of the

treatment effect of radical cystectomy through the creation of *potential outcomes*: two predicted outcomes for each individual patient participant, under the hypothetical assumption that they had been treated with either radical cystectomy or medical management. Next, similar to a propensity score approach, TMLE estimates the probability of treatment with radical cystectomy using either logistic regression, machine learning (LASSO, regression trees, etc.) or super learning approaches.[16] In the third step of the TMLE approach, we use the probabilities obtained in the second step to update the initial estimate of each patient participant's pair of potential outcomes under each treatment group. Specifically, we will generate *targeted* estimates of the potential outcomes with regression models that incorporate both the initial potential outcome estimates and the probability of being treated with radical cystectomy. The average treatment effect is the mean difference between the updated potential outcomes and is interpreted as the causal difference in QOL if all patients had been treated with medical management versus having been treated with radical cystectomy.[16] Because each patient has a pair of potential outcomes under each treatment, subgroup analyses are straightforward. As a secondary sensitivity analysis, we will evaluate these data using propensity score and G-computation methods. As a doubly robust and more efficient analytic approach relative to the comparative secondary approaches, we will use TMLE for presentation of the primary results for this study.

Longitudinal trajectories of the effect of treatment on patient QOL over time will be modeled and characterized using standard linear mixed effects models (primary analysis) or generalized estimating equations (GEE, secondary analysis). We will include covariates in the longitudinal data model to provide treatment effect estimates for subgroups of interest such as those defined by age, comorbidity, caregiver status, tumor severity, and use of investigational agents. For assessment of time-to-event outcomes (e.g., survival), we will use an extension of the TMLE approach appropriate for survival data.[17]

In a comparative study using observational data, one perceived threat to validity is that the types of patients who choose radical cystectomy are inherently different demographically or clinically compared with patients who choose medical management. To evaluate this concern, we examined linked SEER-Medicare data for the years 2004-2011 and identified 2,359 patients with recurrent high-grade NMIBC who underwent at least two courses of BCG and compared them with 551 patients with recurrent high-grade NMIBC who underwent radical cystectomy following at least one course of intravesical BCG. These inclusion criteria matched our anticipated CISTO cohort as rigorously as possible with administrative data.

Aim 2 hypotheses concern the effects of patients' preferences at baseline on treatment choice and future quality of life. Preferences and quality of life will be assessed at baseline and at 12 month follow-up using time tradeoff (TTO) questions, in which patients indicate how many years of life they would be willing to "trade off" in order to experience perfect health instead of their current health (to measure quality of life) or one of three negative health outcomes (to measure health preferences): retained bladder and non-metastasized NMIBC, removed bladder and non-metastasized NMIBC, and metastasized bladder cancer. These questions yield a utility score for each health state. Quality of life will also be assessed using the EQ-5D-5L. Hypothesis 2.1 concerns the relationship between treatment choice and patient preferences at baseline for anticipated health conditions in the future. To test this hypothesis, treatment choice will be modeled as a function of patient preferences for future health states while controlling for patients' current health-related QOL, demographic and other characteristics using logistic regression. Hypothesis 2.2 concerns the relationship between changes to experienced health-related QOL from baseline to 12 months post-enrollment and baseline patient preferences for future health states. To test this hypothesis, health-related QOL at 12 months post-enrollment will be modeled as a function of patient preferences while controlling for patients' health-related QOL at baseline, demographic and other characteristics using beta

regression, as described in Basu 2011,[18] stratified by treatment arm. We anticipate that individuals who survive and complete QOL evaluations at 12 months post-treatment may not be representative of all those initially enrolled. We plan to account for this using an inverse probability weighting approach. Both measures of QOL at 12 months post-enrollment (TTO and EQ-5D-5L) will be used in separate models, each with the corresponding measure at baseline included as a covariate.

To identify key themes for Aim 2 patient and caregiver interviews, interview transcripts will be reviewed for accuracy and de-identified before being uploaded into a qualitative analysis program.[19] Members of the research team will review transcripts and independently generate an initial code list. Through an iterative process of coding, discussion, and reconciliation, coding pairs will propose new codes and identify coding patterns. The coding team will summarize emerging themes for the larger team to review and assess thematic saturation as the point at which additional interviews do not yield more information about decision-making and preferences for recurrent high-grade NMIBC that have failed first-line BCG.

9.3 Missing Data

The CISTO research team aims to maintain the highest level of participant involvement and survey completion; they have achieved over 90% completion rates over similar time periods in a prior study with a similar data collection burden.[11] Because missing data is always a possibility, the DCC will monitor missing data through automated nightly reports, making these available to staff at CISTO practice sites to correct missing data fields, if possible. These reports will also be used to create a plan to prevent missing data in the future a participant or practice site which previously provided incomplete data. Important data elements will be prospectively monitored to examine patterns or reasons for “missingness”.

As a part of treatment effect estimation, the TMLE algorithm provides an integrated approach to incorporating uncertainty that arises due to missing longitudinal data. The mean outcome conditional on observing the outcome may be a biased estimate when missingness is informative. TMLE can reduce this bias when missingness is a function of measured baseline demographic and clinical characteristics. Missing outcome measurements will be accounted for by nonparametrically estimating the missing data mechanism to produce a matrix of missing data probabilities conditioned on baseline demographic and clinical characteristics. The missing data conditional probabilities will then be incorporated into the TMLE estimation procedure during the third step of updating potential outcomes.[13]

When missing data are suspected to be *missing not at random*, any standard analysis of the available cases is likely biased. The bias cannot be corrected since missingness depends on unobserved data and therefore cannot be empirically modelled reliably. We will therefore assess the sensitivity of inferences made from missing data by imputing missing data under both pessimistic and optimistic scenarios and repeating the TMLE algorithm to provide bounds on the statistical uncertainty. The characteristics of non-responders will be summarized in our final report and we will present the heterogeneity of the treatment effect due to missing data.

9.4 Sample Size and Statistical Power

We aim to recruit from a heterogeneous patient population that will allow for rich and broadly applicable estimation of outcomes and experiences after NMIBC that recurs after first-line BCG. **We will enroll up to 900 participants across a broad geographic recruitment area.** This cohort would represent the largest pragmatic study of recurrent high-grade NMIBC patients to date. The total sample size for CISTO is primarily motivated by statistical power considerations for **Aim 1**, comparing QOL between patients treated with medical management versus radical cystectomy. Presently, there are no guiding documents

on directly using TMLE in the calculation of statistical power for designing an observational study. The variance of the treatment effect estimate—and thus power—is a complicated function of the number treated in each group, the potential and observed outcomes, and the covariate-based probabilities of treatment with radical cystectomy. The 2:1 recruitment ratio of medical management to radical cystectomy patients at each CISTO site will ensure at least a 33% rate of radical cystectomy in the CISTO study population. However, based on actual rates of enrollment of cystectomy patients, we motivated the study sample size of 572 using 40.9% cystectomy patients in a standard two-sample repeated measures approach that will be less efficient than the analytic methods used at the time of data analysis.

Statistical power was fixed at 0.80 throughout and we considered two-sided tests with alpha=0.05 indicating statistical significance, with no adjustment for multiple comparisons for the primary question of interest. Correlation between repeated measures was assumed to be 0.3, an estimate we have observed in numerous other QOL studies. Minimal detectable effect sizes (units of QOL standard deviation) are reported in **Table 3** below for Aim 1 within subgroups with varying prevalence.

Table 3. Minimal Detectable Effect Sizes

		Subgroups		
	Overall	50%	30%	20%
Aim 1: Patient QOL	0.24	0.34	0.44	0.54

Aim 2 is largely descriptive in nature and no statements of statistical power were considered. With recruitment of 572 patient participants, anticipating 235 radical cystectomy patients and 337 patients treated with medical management in Aim 1 and conservatively allowing for 10% missing data, this study has >0.80 statistical power to detect small differences in QOL between treatment approaches (Cohen's d = 0.24)[20] that are well below the minimally important difference of 0.5. Assuming a similar balance of treatments within subgroups, this study has >0.80 power to detect moderate but clinically important effect sizes (Cohen's d = 0.43) within subgroups as small as 30% of the study cohort. From SEER, large treatment effect differences would be detectable in subgroups of gender (20% female), age (52% over 75), comorbid health conditions (22% Charlson Index 1 or greater), and CIS (25%). We will plan a subgroup analysis for patients receiving investigational agents as part of a phase III clinical trial if at least 10% of participants in the medical management arm are in a trial. To inform power calculations for our secondary outcomes of bladder cancer-specific mortality, we used data from a microsimulation model demonstrating low anticipated 1-year mortality event rates (0.8% for radical cystectomy, 3.6% for continued medical management). We would have 69% power to detect a similar difference in one-year mortality due to bladder cancer and 80% power to evaluate the difference between study arms in 2-year bladder cancer-specific mortality.

10.0 Data and Safety Monitoring Plan

Given the minimal risk nature of this observational study, monitoring will be conducted by the CISTO Executive Committee. The CISTO Executive Committee consists of the chair, the co-PIs, the director of the Data Coordinating Center (DCC), the director of the Clinical Coordinating Center (CCC), and stakeholder representatives covering expertise in clinical epidemiology, biostatistics, and urologic oncology. The CISTO Executive Committee meets monthly and time during one Executive Committee

meeting per quarter will be set aside to review any data and safety events and procedures and to determine recommendations for these events and procedures as appropriate. This briefing will cover the following procedures:

- Monitoring study safety
- Minimizing research-associated risk
- Protecting the confidentiality of participant data
- Identifying, reviewing, and reporting adverse events (AEs) and serious adverse events (SAEs) and unanticipated problems to the applicable IRB(s) or other monitoring bodies
- Procedures for ensuring compliance with the DSMP and the requirements for reporting across research study sites.

10.1 SAE Reporting

Participants will be monitored for SAEs throughout their study surveillance period (enrollment through up to four-year follow-up). The occurrence of an SAE may come to the attention of study personnel during study follow-up phone interview, by UW study staff personnel conducting follow-up, or by a study participant calling the study team or presenting for medical care. If an SAE comes to the attention of study personnel via patient, caregiver, or family report, the site research coordinator will be notified that an SAE form needs to be completed. The site research coordinator will then use the EMR to fill out the SAE form. The site PI will then complete the form and sign the form. The SAE form will then be submitted to the DCC who will provide it to the CISTO Executive Committee. The CISTO Executive Committee will review the SAE form and ask the site PI for additional clarification or specification as they see necessary.

SAEs are defined as one of the following conditions:

1. Death during the period of protocol-defined surveillance;
2. Life-threatening event related to the treatment or significant disability/incapacity related to the treatment;
3. Inpatient hospitalization (other than for cystectomy);
4. Prolonged hospitalization following cystectomy (14 days or more).

All SAEs will be:

1. Recorded on the SAE case report form;
2. Followed until satisfactory resolution or until the study investigators deem the event to be chronic or the participant to be stable;
3. Reported to the study investigators, the project managers, and site IRBs per their reporting guidelines.

CISTO is an observational study using procedures or treatments with well-established risk profiles. There is no reason to suspect the risk profiles in the proposed study population would differ. The number of adverse events and related unexpected serious adverse events (SAEs) will be summarized descriptively by arm, by grade and body system. The proportion of participants experiencing each toxicity will be summarized by maximum National Cancer Institute's Common Terminology Criteria for Adverse Events (NCI CTCAE) grade[21] experienced, overall and by arm.

The local and systemic side effects associated with intravesical therapy will be graded according to the International Bladder Cancer Group's (IBCG) recommendations[22] (percentage indicates incidence rate):

- Cystitis noninfective (33-80%)

- Hematuria (up to 90%)
- Urinary tract obstruction (rare)
- Urinary tract infection
- Prostate infection (1-3%)
- Epididymoorchitis (0.2-10%)
- Rash maculo-papular (up to 19%)
- Malaise (30.5%)
- Fever (5-20%)
- Allergic reactions (rare)
- Myelosuppression (rare – anemia/neutropenia/thrombocytopenia)
- Pelvic pain
- Renal colic

The systemic side effects associated with immunotherapy (pembrolizumab) will be graded and reported as per previous reports[23–25] (percentage indicates incidence rate):

- Fatigue (11-29%)
- Pruritus (10-22.7%)
- Hypothyroidism (7-21.1%)
- Hyperthyroidism (5-11.9%)
- Arthralgia (4-22.1%)
- Pneumonitis (3-4.1%)
- Dry mouth (3%)
- Asthenia (2-10.2%)
- Aspartate aminotransferase increased (2%)
- Myalgia (2%)
- Cough
- Headache
- Increase in blood creatinine level

Operative radical cystectomy is a major operation that has a number of recognized complications and a low risk of death (less than 2 in 100). Expected adverse events will be graded using the Clavien Dindo classification[26] and reported as per previous reports[27,28] (percentage indicates incidence rate):

- Gastrointestinal (29%): ileus, small intestinal obstruction, constipation, enterocolitis infectious, upper gastrointestinal hemorrhage, lower gastrointestinal hemorrhage, vomiting, small intestinal anastomotic leak, diarrhea, colonic obstruction, dehydration, and nausea.

- Infectious (25%): abdominal infection, urinary tract infection, sepsis, gastritis and cholecystitis.
- Wound (15%): seroma, wound infection and wound dehiscence.
- Genitourinary (11%): acute kidney injury, urinary tract obstruction, ureteric anastomotic leak, urethral anastomotic leak, urinary fistula, urinary retention, parastomal hernia, gastrointestinal stoma necrosis and hematuria.
- Cardiac (11%): ventricular arrhythmia, myocardial infarction, hypertension, heart failure, chest pain-cardiac and hypotension.
- Pulmonary (9%): atelectasis, lung infection, dyspnea, pneumothorax and pleural effusion.
- Bleeding (9%): anemia and postoperative hemorrhage.
- Thromboembolic (8%): thromboembolic event and phlebitis.
- Neurological (5%): peripheral motor neuropathy, transient ischemic attacks, delirium, vertigo, depressed level of consciousness and seizure.

11.0 Data Quality and Performance Management

11.1 Site Training

Prior to study initiation and enrollment, all clinicians and staff who will be recruiting patients with recurrent high-grade NMIBC and their caregivers will complete study-specific training; a training log will be kept locally at each participating site.

The CCC will be responsible for maintaining administrative, clinical, and operational coordination for all aspects of the study. All site training and operational activities will be monitored and coordinated through the CCC. This includes training of research coordinators and site leaders, maintenance of data abstraction quality, and completeness of data.

11.2 Quality Management Plan

The quality management plan (QMP) will include verification that sites have appropriately met the requirements to assure quality of all study-related activities. Each site will be responsible for maintaining accurate and complete patient charts and informed consent documentation. The QMP is in place to verify good practice.

The CCC and DCC will regularly review participant enrollment, reasons for exclusion, participant demographics, and follow-up rates by site in order to assess proximity to enrollment targets, site performance, and protocol adherence.

The DCC will set up in-line data quality checks within the data entry system, which will check for missing information, inconsistencies, and logic errors. The CCC and DCC will monitor this information on a regular basis. In addition, the DCC will perform quality assurance checks by running regular data quality reports. These reports include missing values for required fields, incorrect data type, range checks, outliers, hidden fields that contain values, and multiple-choice fields with invalid values. Values that need to be corrected will be brought to the attention of the research staff at that site. The DCC and CCC will investigate any discrepancies or unexpected values that were discovered through the regular report or through visual inspection.

Research staff from the CCC will be responsible for regular remote monitoring of each site. This will include review of participant enrollment forms, patient reported outcomes data entry, EMR data abstraction, and any AEs or SAEs. Source documentation may be uploaded in designated REDCap fields. Regular meetings with the research site leads at each site will be held via phone to relay any concerns or walk through issues. Retraining will be available through the CCC if needed.

For sites that are found to have several inconsistencies and errors in data quality and completeness, or an increased number of protocol deviations compared to other sites, monitoring visits will be scheduled and conducted as needed.

11.3 Performance Management Plan

Site study leadership teams will review enrollment rates and any outstanding needs or concerns with the CCC on a regular basis. The purpose of these individual site meetings is to monitor site progress against anticipated and expected site enrollment targets. If screening/recruitment goals are not being met, the CCC will implement the following Performance Management Plan, consisting of:

1. Site Progress Call to review and discuss:
   - i. Site specific screening and enrollment rates
   - ii. Missed participants, study refusals
   - iii. EMR abstraction completion rates
   - iv. Statement of work and milestones review
   - v. Support or training needs for the site from the CCC
   - vi. Plan of action to address enrollment shortfalls
2. Interval Check-In Call:
   - i. Site specific screening
   - ii. Plan of action results
3. Follow-up Call(s) as needed:
   - i. Continued training
   - ii. In-person site visit (as needed)

Site performance will be monitored on a regular basis by the CCC and concerns will be elevated to the CISTO Executive Committee. If a site consistently fails to meet expectations in comparison to the average study site, suspension or termination of the site subcontract may be necessary.

11.4 Protocol Violations

A protocol violation is any non-compliance with the clinical trial protocol or Good Clinical Practices. The noncompliance may be either on the part of the participant, the investigator, or the study site staff and most often is related to study enrollment. Anticipated protocol violations are 1) Participant enrolled but did not meet inclusion criteria, 2) Participant enrolled but exclusion criteria present, 3) Consent not obtained in accordance with IRB guidelines. Participants enrolled and later found to be ineligible may be followed depending on information available at enrollment.

All violations from the protocol will be addressed in study participant source documents. A record of the violation will be maintained in the regulatory file, as well as in the participant's study chart. Protocol

violations will be sent to the local IRB per their guidelines. The site investigator/study staff will be responsible for knowing and adhering to their IRB requirements.

12.0 Protection of Human Participants

Prisoners will be excluded from CISTO study participation.

The primary risks of this study are loss of patient privacy, loss of confidentiality, and study burden. Loss of confidentiality could occur if the study database were breached. This risk will be low as a result of numerous steps to protect confidentiality. Approaching patients about enrollment in the clinic poses a risk to privacy. We anticipate this risk to be low. Patients will be approached in a private clinic room. To avoid threats of coercion, providers will be educated but not directly involved with recruitment of participants. All participants may be at higher risk for psychological discomfort or anxiety due to perceived or actual additional burdens related to study participation. Questions about quality of life and bothersome symptoms may be uncomfortable for some participants, but are also part of standard of care during many routine clinic visits. Thus, we expect the risks of discomfort or anxiety related to study procedures to be low. It is unlikely there will be direct, short-term benefits to individual participants in this study. The long-term benefit arises from the potential to improve decision-making for patients deciding on treatment following a diagnosis of recurrent high-grade bladder cancer.

Informed consent is a process that is initiated prior to the individual's agreeing to participate in the study and continuing throughout the individual's study participation. Extensive discussion of risks and possible benefits of both treatment options will be provided to the participants using standardized materials, which can include an informational video. Consent forms describing in detail the study procedures and risks will be given to the participant and documentation of informed consent will be required prior to completing study assessments.

Consent forms will be IRB-approved and the participant will be given sufficient time to read and review the document and discuss with their caregiver, family member, friend, or legal representative. If they require assistance to reach their caregiver, family member, friend, or legal representative, such as the use of a telephone, that will be facilitated. After this, they will be specifically asked if they have any questions or concerns, which will be addressed, or would like more time to consider their participation, which will also be provided. The study investigator or coordinator will explain the research study to the participant and answer any questions that may arise. The participants will sign the informed consent prior to completing any study related activities. The participants may withdraw consent at any time throughout the course of the study. A copy of the informed consent will be given to the participants for their records. The rights and welfare of the participants will be protected by emphasizing to them that they will still be able to receive medical care at the facility if they decline to participate in this study.

Participant confidentiality is strictly held in trust by the participating investigators and their staff.

13.0 Ancillary Studies

An ancillary study subcommittee within the CCC will work with interested investigators, representatives of PCORI and CISTO stakeholders to coordinate, seek funding for, facilitate and plan ancillary studies that may be linked to the CISTO trial.

14.0 References

1. Siegel RL, Miller KD, Wagle NS, Jemal A. Cancer statistics, 2023. *CA: A Cancer Journal for Clinicians*. 2023;73(1):17-48. doi:10.3322/caac.21763

2. Carter AJR, Nguyen CN. A comparison of cancer burden and research spending reveals discrepancies in the distribution of research funding. *BMC Public Health*. 2012;12:526. doi:10.1186/1471-2458-12-526

3. Kamat AM, Sylvester RJ, Böhle A, et al. Definitions, End Points, and Clinical Trial Designs for Non-Muscle-Invasive Bladder Cancer: Recommendations From the International Bladder Cancer Group. *J Clin Oncol*. 2016;34(16):1935-1944. doi:10.1200/JCO.2015.64.4070

4. Kaufman DS, Shipley WU, Feldman AS. Bladder cancer. *Lancet*. 2009;374(9685):239-249. doi:10.1016/S0140-6736(09)60491-8

5. Power NE, Izawa J. Comparison of Guidelines on Non-Muscle Invasive Bladder Cancer (EAU, CUA, AUA, NCCN, NICE). *Bladder Cancer*. 2016;2(1):27-36. doi:10.3233/BLC-150034

6. Sylvester RJ, van der Meijden APM, Oosterlinck W, et al. Predicting recurrence and progression in individual patients with stage Ta T1 bladder cancer using EORTC risk tables: a combined analysis of 2596 patients from seven EORTC trials. *Eur Urol*. 2006;49(3):466-5; discussion 475. doi:10.1016/j.eururo.2005.12.031

7. Flum DR, Alfonso-Cristancho R, Devine EB, et al. Implementation of a “real-world” learning health care system: Washington State’s Comparative Effectiveness Research Translation Network (CERTAIN). *Surgery*. 2014;155(5):860-866. doi:10.1016/j.surg.2014.01.004

8. Morris NS, MacLean CD, Chew LD, Littenberg B. The Single Item Literacy Screener: Evaluation of a brief instrument to identify limited reading ability. *BMC Fam Pract*. 2006;7(1):21. doi:10.1186/1471-2296-7-21

9. Reed O, Jubber I, Griffin J, et al. Occupational bladder cancer: A cross section survey of previous employments, tasks and exposures matched to cancer phenotypes. Mukherjee A, ed. *PLoS ONE*. 2020;15(10):e0239338. doi:10.1371/journal.pone.0239338

10. Aaronson NK, Ahmedzai S, Bergman B, et al. The European Organization for Research and Treatment of Cancer QLQ-C30: a quality-of-life instrument for use in international clinical trials in oncology. *Journal of the National Cancer Institute*. 1993;85(5):365-376. doi:10.1093/jnci/85.5.365

11. Gilbert SM, Wood DP, Dunn RL, et al. Measuring health-related quality of life outcomes in bladder cancer patients using the Bladder Cancer Index (BCI). *Cancer*. 2007;109(9):1756-1762. doi:10.1002/cncr.22556

12. de Souza JA, Yap BJ, Wroblewski K, et al. Measuring financial toxicity as a clinically relevant patient-reported outcome: The validation of the COmprehensive Score for financial Toxicity (COST). *Cancer*. 2017;123(3):476-484. doi:10.1002/cncr.30369

13. Gruber S, van der Laan MJ. A targeted maximum likelihood estimator of a causal effect on a bounded continuous outcome. *Int J Biostat*. 2010;6(1):Article 26. doi:10.2202/1557-4679.1260

14. Rosenblum M, van der Laan MJ. Targeted maximum likelihood estimation of the parameter of a marginal structural model. *Int J Biostat*. 2010;6(2):Article 19. doi:10.2202/1557-4679.1238

15. Schuler MS, Rose S. Targeted maximum likelihood estimation for causal inference in observational studies. *Am J Epidemiol*. 2017;185(1):65-73. doi:10.1093/aje/kww165

16. Pirracchio R, Petersen ML, van der Laan M. Improving propensity score estimators' robustness to model misspecification using super learner. *Am J Epidemiol*. 2015;181(2):108-119. doi:10.1093/aje/kwu253

17. Stitelman OM, Gruttola VD, Laan MJ van der. A General Implementation of TMLE for Longitudinal Data Applied to Causal Inference in Survival Analysis. *The International Journal of Biostatistics*. 2012;8(1). doi:10.1515/1557-4679.1334

18. Basu A, Manca A. Regression estimators for generic health-related quality of life and quality-adjusted life years. *Med Decis Making*. 2012;32(1):56-69. doi:10.1177/0272989X11416988

19. ATLAS.ti. ATLAS.ti. Accessed April 7, 2023. https://atlasti.com/research-hub/citing-atlas-ti-in-your-research

20. Sawilowsky SS. New Effect Size Rules of Thumb. *J Mod App Stat Meth*. 2009;8(2):597-599. doi:10.22237/jmasm/1257035100

21. Common Terminology Criteria for Adverse Events (CTCAE). Published online 2017.

22. Clinical Practice Recommendations for the Prevention and Management of Intravesical Therapy–Associated Adverse Events.

23. Balar AV, Kamat AM, Kulkarni GS, et al. Pembrolizumab monotherapy for the treatment of high-risk non-muscle-invasive bladder cancer unresponsive to BCG (KEYNOTE-057): an open-label, single-arm, multicentre, phase 2 study. *The Lancet Oncology*. 2021;22(7):919-930. doi:10.1016/S1470-2045(21)00147-9

24. Choueiri TK, Tomczak P, Park SH, et al. Adjuvant Pembrolizumab after Nephrectomy in Renal-Cell Carcinoma. *N Engl J Med*. 2021;385(8):683-694. doi:10.1056/NEJMoa2106391

25. Bellmunt J, de Wit R, Vaughn DJ, et al. Pembrolizumab as Second-Line Therapy for Advanced Urothelial Carcinoma. *N Engl J Med*. 2017;376(11):1015-1026. doi:10.1056/NEJMoa1613683

26. Dindo D, Demartines N, Clavien PA. Classification of Surgical Complications. *Ann Surg*. 2004;240(2):205-213. doi:10.1097/01.sla.0000133083.54934.ae

27. Shabsigh A, Korets R, Vora KC, et al. Defining Early Morbidity of Radical Cystectomy for Patients with Bladder Cancer Using a Standardized Reporting Methodology. *European Urology*. 2009;55(1):164-176. doi:10.1016/j.eururo.2008.07.031

28. Tan WS, Lamb BW, Tan MY, et al. In-depth Critical Analysis of Complications Following Robot-assisted Radical Cystectomy with Intracorporeal Urinary Diversion. *European Urology Focus*. 2017;3(2):273-279. doi:10.1016/j.euf.2016.06.002
